# Progression-free survival 2 (PFS2) as a surrogate endpoint for overall survival (OS) in breast cancer randomized controlled clinical trials

**DOI:** 10.1016/j.esmoop.2026.106062

**Published:** 2026-01-27

**Authors:** P. Filis, N. Filis, T. Foukakis, A. Matikas

**Affiliations:** 1 Department of Oncology/Pathology, Karolinska Institutet, Stockholm, Sweden; 2 University of Ioannina School of Medicine, Ioannina, Greece; 3 Breast Center, Karolinska Comprehensive Cancer Center and Karolinska University Hospital, Stockholm, Sweden

**Keywords:** progression-free survival 2, PFS2, overall survival, surrogate endpoints, breast cancer

## Abstract

**Background:**

Overall survival (OS) is the gold-standard endpoint in oncology trials but often requires prolonged follow-up. Progression-free survival 2 (PFS2) has been proposed as an intermediate surrogate. While prior analyses report sufficient correlation of PFS2 with OS, no study has specifically evaluated its surrogacy in breast cancer.

**Materials and methods:**

A systematic search of Medline, Web of Science, and Cochrane Library was conducted to identify randomized controlled trials (RCTs) in breast cancer reporting both PFS2 and OS. Trial-level associations between log-transformed hazard ratios for PFS2 and OS were examined using sample size-weighted linear regression. Surrogacy strength was quantified using the coefficient of determination (*R*^2^) and Pearson correlation coefficient (*r*) with 95% bootstrap confidence intervals (CIs).

**Results:**

Eighteen RCTs including 9617 patients were analyzed. The correlation between PFS2 and OS was strong (*r* = 0.714, 95% CI 0.204-0.893, *R*^2^ = 0.509). Validation using long-term OS data showed a moderate but weaker correlation (*r* = 0.552, 95% CI –0.12 to 0.897, *R*^2^ = 0.305). Surrogacy was stronger in trials with PFS2 maturity ≥55% (*r* = 0.93, *R*^2^ = 0.864) versus <55% (*r* = –0.652, *R*^2^ = 0.425), and in those with OS information fraction ≥75% (*r* = 0.931, *R*^2^ = 0.866) versus <75% (*r* = 0.625, *R*^2^ = 0.391). Correlation was similar across OS maturity subgroups (<40%: *r* = 0.677, 40%-60%: *r* = 0.702).

**Conclusion:**

PFS2 demonstrated a context-dependent association with OS, with stronger correlations observed only at higher levels of PFS2 maturity and OS information fraction.

## Highlights

• PFS2 shows a moderate and context-dependent association with OS across 15 randomized trials.
• Higher PFS2 maturity (≥55%) yields stronger OS correlation (*r* = 0.93).
• Improved surrogacy is observed with OS information fraction ≥75% (*r* = 0.931).
• Consistent surrogacy is found across treatment lines and OS maturity levels.

## Introduction

Traditionally, overall survival (OS) has been regarded as the most definitive and clinically meaningful endpoint in oncology trials, provided that patient quality of life is not compromised.^1^ As a direct measure of therapeutic benefit, OS is unambiguous and inherently free from ascertainment bias, given that death constitutes an objective event. In alignment with this, OS has been frequently characterized as the ‛gold-standard’ endpoint in cancer clinical trials.^2^ Despite its advantages, OS as an endpoint is often associated with substantial logistical challenges.^3^ Demonstrating a survival benefit frequently necessitates prolonged follow-up, rendering OS an impractical primary endpoint in studies involving indolent neoplasms or maintenance strategies.^3^ These delays increase the cost, complexity, and duration of clinical development, potentially limiting timely access to new treatments.^1^

To facilitate more efficient drug development, surrogate endpoints have been proposed. Progression-free survival, a widely used endpoint, demonstrates inconsistent correlation with OS across tumor types.^4^^,^^5^ Although initial therapies may delay progression, they may also contribute to the emergence of more aggressive and therapy-resistant tumor clones following treatment.^6^ Furthermore, progression-free survival (PFS) captures disease control only during the study treatment period while subsequent anticancer therapies are often delivered outside the context of randomization or stratification, introducing heterogeneity that can confound the assessment of a true survival advantage from the experimental agent.^4^

In recognition of these limitations, the European Medicines Agency (EMA) has advocated for the use of PFS2, as an intermediate endpoint when OS cannot be feasibly assessed.^7^ PFS2 is defined as the interval from randomization (or registration in non-randomized studies) to objective progression on subsequent therapy or death from any cause, whichever occurs first.^8^ In some cases, time on next-line therapy might be used as a proxy for PFS2.^8^ In a recent analysis of advanced cancer randomized trials PFS2 was found to have improved correlation with OS compared with PFS.^5^ However, these data pertain to a mixed population of cancers, primarily lung cancer, without subgroup analysis by tumor type. Moreover, a high correlation of PFS2 with OS was shown for lung cancer from a previous report.^9^ Currently, there is no published study that assesses the use of PFS2 as a surrogate for OS in breast cancer trials, while prior literature did not support the surrogacy of PFS for OS in the advanced setting.^10^^,^^11^

PFS2 is increasingly evaluated as an endpoint in breast cancer clinical trials, where multiple lines of therapy and prolonged OS complicate the use of OS as a primary outcome.^12^^,^^13^ This study aimed to systematically review the use of PFS2 in breast cancer trials and to assess its validity as a surrogate for OS, with particular attention to factors that may influence the strength of this association.

## Materials and methods

This study is reported in accordance with the Reporting of Surrogate Endpoint Evaluation using Meta-analyses (ReSEEM) guidelines,^14^ and the Preferred Reporting Items for Systematic Reviews and Meta-Analyses (PRISMA) reporting guideline.^15^ The study protocol was registered with the international prospective register of systematic reviews, PROSPERO (identifier CRD420251089417).

### Search strategy

A systematic literature search was conducted in the Medline, Web of Science, and Cochrane Library databases from database inception to 15 June 2025. The search terms included ‛progression-free survival 2’, ‛PFS2’, ‛second progression’, and ‛breast cancer’. Two independent reviewers (PF, NF) screened titles and abstracts of all identified records for eligibility. Full texts were retrieved for studies deemed potentially relevant. Discrepancies were resolved through discussion or consultation with a third reviewer (AM). To ensure completeness, the reference lists of included studies and recent relevant reviews were also screened.

### Study selection and data extraction

Eligibility was defined according to the PICOS framework as follows: Population: patients with breast cancer; Intervention/Comparator: any therapeutic intervention compared with any comparator; Outcomes: studies reporting data on both PFS2 and OS; Study design: randomized controlled trials (RCTs). Non-randomized studies, including prospective cohort studies, retrospective analyses, and single-arm trials, were excluded. Trials providing OS data from long-term follow-up were also considered included.

Data extraction was carried out independently by two reviewers (PF, NF), with discrepancies resolved through consultation with a third reviewer (AM). Extracted data included: first author, publication year, trial name, study population, number of prior lines of treatment, intervention and control regimens, subsequent therapies, median follow-up (months), number of patients in each arm, PFS2 definition, whether PFS2 was predefined in the protocol (yes/no), PFS2 endpoint designation (primary, secondary, exploratory), hazard ratio (HR) for PFS2 with 95% confidence intervals (CIs), median PFS2 (months) for each group, HR for OS with 95% CIs, median OS (months) for each group, and HR for PFS1 with 95% CIs. For studies reporting long-term follow-up, updated OS data and the percentage of patients in the control group who received the intervention treatment were also recorded. For trials with multiple follow-up publications, data from the publication with the longest follow-up were extracted.

Consistent with prior surrogate endpoint validation studies, no formal risk-of-bias assessment was carried out, as the objective was to evaluate trial-level associations rather than to synthesize pooled efficacy estimates.^5^^,^^16^

### Statistical analysis

All analyses were conducted at the trial level using data extracted from published RCTs in breast cancer. The primary objective was to evaluate whether PFS2 could serve as a surrogate endpoint for OS. For the primary analysis, we examined the association between PFS2 and OS using linear regression, regressing log-transformed HRs [log(HR OS)] on log(HR PFS2) [model: log(HR OS) = β0 + β1 × log(HR PFS2) + ε]. Analyses were weighted by the total sample size of each trial, in accordance with published literature on surrogate endpoint validation.^14^ We calculated the coefficient of determination (*R*^2^) to assess the proportion of variance in OS explained by PFS2. An *R*^2^ of ≥0.7 is conventionally considered indicative of trial-level surrogacy in oncology.^14^ To further characterize the association, we calculated the sample size-weighted Pearson correlation coefficient (*r*) and its 95% CI using bootstrap resampling. We interpreted the strength of the correlation as follows: *r* < 0.5 was considered poor to fair, *r* in the range of 0.5-0.7 as moderate, and *r* > 0.7 as strong, in line with commonly used thresholds in clinical medicine research.^17^ *P* values for the regression slope were reported using a two-sided significance threshold of *P* < 0.05.

Secondary analyses included the following: (i) the association between PFS2 and OS in trials that provided updated OS data during study follow-up; (ii) the association between PFS2 and the change in OS treatment effect over time (for studies that provided OS data both on time point of PFS2 measurement as well as on long-term follow-up), defined as Δlog(HR OS), assessed using Spearman’s *ρ*; (iii) the correlation between median PFS2 and median OS in months across all trial arms, evaluated using Spearman’s rank correlation coefficient (*ρ*); (iv) the correlation between PFS2 and OS in breast cancer subtypes; and (v) the correlation between PFS1 and OS.

Subgroup analyses were conducted to assess whether surrogate validity varied according to OS maturity, OS information fraction, PFS2 maturity, the proportion of patients in the control group who were exposed to experimental treatment, as well as the line of treatment at study initiation. Maturity was defined as the proportion of patients in the intention-to-treat population who had experienced the event of interest at the time of reporting (number of events/number of patients). Information fraction referred to the proportion of the total planned number of events that had occurred at the time of analysis (number of events/number of planned events). Number of planned events was pre-specified by protocol and in cases where multiple OS analyses were planned, the number of events corresponding to the final planned analysis was used.

The robustness of the primary analysis was assessed through a leave-one-out sensitivity analysis, in which the linear regression was repeated iteratively after excluding one trial at a time. For each iteration, the slope, *R*^2^, Pearson correlation coefficient (*r*) with 95% CI, and *P* value for the regression slope were recorded. All analyses were conducted using R (version 4.2.2).

## Results

**Figure 1:**
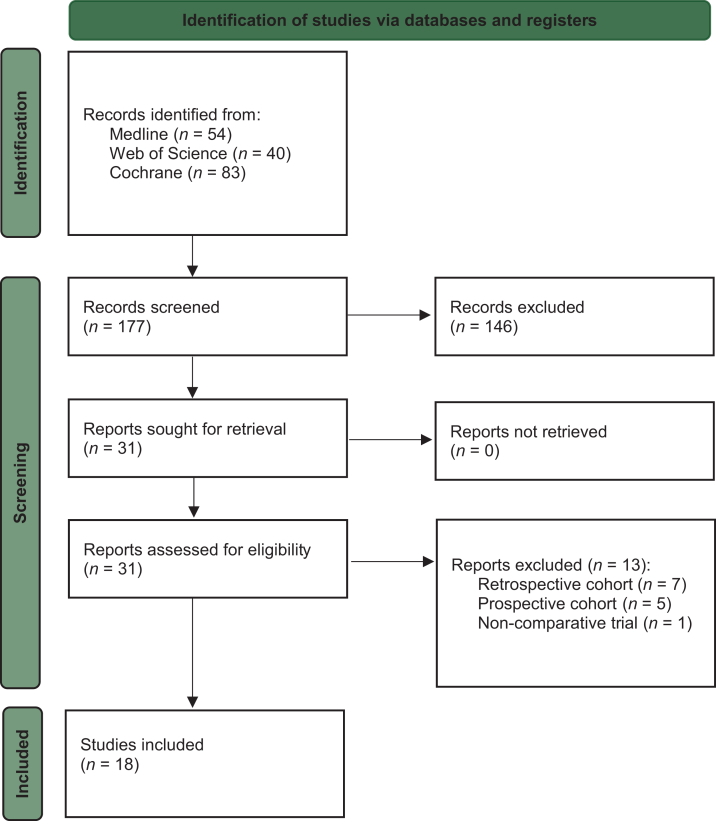
Study flow chart.

A total of 18 RCTs for breast cancer were included in this study, following a systematic search of the Medline, Web of Science, and Cochrane Library databases (Figure 1). A list of excluded studies, along with the reason for exclusion, are presented in Supplementary Table S1, available at https://doi.org/10.1016/j.esmoop.2026.106062. Of the 18 included RCTs, PFS2 data were available in the original full-text publication for 15 studies,^12^^,^^18^, ^19^, ^20^, ^21^, ^22^, ^23^, ^24^, ^25^, ^26^, ^27^, ^28^, ^29^, ^30^, ^31^, ^32^, ^33^ while for 3 trials PFS2 data were extracted from published congress abstracts.^13^^,^^34^^,^^35^ In addition, 10 trials reported updated OS outcomes following long-term follow-up.^36^, ^37^, ^38^, ^39^, ^40^, ^41^, ^42^, ^43^, ^44^, ^45^

### Study characteristics

Characteristics of the included studies are summarized in Table 1. The trials collectively enrolled 9617 patients with advanced or metastatic breast cancer, with publications spanning from 2014 to 2025. Of the 18 included trials, 11 enrolled patients with hormone receptor-positive, human epidermal growth factor receptor 2 (HER2)-negative disease, 4 enrolled patients with HER2-positive disease, and 3 enrolled patients with HER2-negative disease (Table 1). The three HER2-negative trials included mixed populations of hormone receptor-positive HER2-negative and triple-negative breast cancer, without consistent reporting of stratified PFS2 by subtype. Among the 11 hormone receptor-positive HER2-negative trials, 10 evaluated endocrine-based treatment strategies, whereas one assessed an antibody–drug conjugate-based regimen (Supplementary Table S2, available at https://doi.org/10.1016/j.esmoop.2026.106062). Only 6 out of 18 trials enrolled patients who were not previously treated for the advanced/metastatic setting, while the remaining studies included participants with one or more prior lines of systemic therapy. Details on the investigational and control treatments of the included studies, along with HRs and corresponding 95% CIs, for PFS1, PFS2, and OS, are presented in Supplementary Tables S2 and S3, available at https://doi.org/10.1016/j.esmoop.2026.106062. The percentage of patients in the control group that received the intervention arm treatment in the second line ranged from 10.8% to 56.3% (Supplementary Table S3, available at https://doi.org/10.1016/j.esmoop.2026.106062).

**Table 1:** Characteristics of the included studies

Trial	Year	Breast cancer type	Prior lines of therapy	PFS2 included in protocol	PFS2 endpoint	*N* Intervention	*N* Control	Median follow-up (months)	OS maturity (%)	OS information fraction (%)	PFS2 maturity (%)	Long-term OS data available	Refs.
SERENA-6	2025	HR-positive HER2-negative ABC	1	Yes	Secondary	157	158	12.6	39/315 (12.4)	39/165 (24)	85/315 (27)	No	^12^
DESTINY-Breast09	2025	HER2-positive ABC or MBC	0	NR	NR	383	387	29.2	126/770 (16.4)	NR	193/770 (25)	No	^13^
TROPION-Breast01	2025	HR-positive HER2-negative MBC	1 or 2	Yes	Secondary	365	367	10.8	171/732 (23.4)	171/444 (38.5)	238/732 (32.5)	No	^18^
SONIA	2024	HR-positive HER2-negative ABC	0	Yes	Primary	524	526	37.3	372/1050 (35)	NR	591/1050 (56.3)	No	^19^
INAVO120	2024	PIK3Cam HR-positive HER2-negative ABC or MBC	0	Yes	Exploratory	161	164	21.3	97/325 (29.8)	97/153 (63.4)	NR	Yes	^20^^,^^34^^,^^36^
CAPItello-291	2023	HR-positive HER2-negative ABC	1 or 2	Yes	Secondary	355	353	13	195/708 (27.5)	195/492 (39.6)	383/708 (54.1)	No	^21^^,^^35^
DESTINY-Breast03	2023	HER2-positive MBC	>1	Yes	Exploratory	261	263	28.4	169/524 (32.3)	169/250 (67.6)	NR	Yes	^22^^,^^37^
DESTINY-Breast02	2023	HER2-positive MBC	>1	Yes	Exploratory	406	202	21.5	230/608 (37.8)	230/434 (53)	NR	No	^23^
Pearl	2022	HR-positive HER2-negative MBC	1	No	Exploratory (*post hoc*)	149	299	28	171/300 (57)	171/152 (112)	230/305 (75.4)	No	^24^
MONALEESA-3	2021	HR-positive HER2-negative ABC	0	Yes	Exploratory	237	128	56.3	364/726 (50.1)	364/273 (133)	428/726 (59)	Yes	^25^^,^^38^
MONARCH-3	2021	HR-positive HER2-negative ABC	0	No	Exploratory	328	165	39	NR	NR	NR	Yes	^26^^,^^39^
MONARCH-2	2020	HR-positive HER2-negative ABC	1	No	Exploratory	446	223	47.7	338/669 (52.9)	338/441 (76.7)	457/669 (68.3)	No	^27^
BROCAD3	2020	HER2-negative ABC or MBC	2	No	Secondary	337	172	35.7	254/509 (49.9)	254/357 (71.1)	310/509 (60.9)	Yes	^28^^,^^40^
MONALEESA-7	2019	HR-positive HER2-negative ABC	1	No	Exploratory	335	337	34.6	192/672 (28.6)	192/252 (76.2)	287/672 (42.7)	Yes	^29^^,^^41^
PALOMA-3	2018	HR-positive HER2-negative ABC	>1	No	Exploratory	347	174	44.8	310/521 (59.5)	310/198 (157)	NR	Yes	^30^^,^^42^
OlympiAD	2017	HER2-negative MBC	1	Yes	Secondary	205	97	14.5	140/302 (46.4)	140/190 (73.7)	NR	Yes	^31^^,^^43^
TANIA	2014	HER2-negative locally recurrent or MBC	0	Yes	Primary	247	247	15.9	200/494 (40.5)	NR	407/494 (82.4)	Yes	^32^^,^^44^
VITAL	2014	HER-positive MBC	1	No	Exploratory	75	37	NR	35/112 (31.2)	NR	NR	Yes	^33^^,^^45^

ABC, advanced breast cancer; HER2, human epidermal growth factor receptor 2; HR, hormone receptor; MBC, metastatic breast cancer; NR, not reported; OS, overall survival; PFS2, progression-free survival 2; PIK3CAm, phosphatidylinositol-4,5-bisphosphate 3-kinase catalytic subunit alpha mutant.

PFS2 was included in the study protocol in 13 out of 18 trials (72.2%). Overall, 2 trials (11.1%) specified PFS2 as a primary endpoint, 5 (27.7%) as a secondary, and 10 (55.6) as exploratory (Table 1). The PFS2 definitions were mostly consistent among trials and are included in Supplementary Table S2, available at https://doi.org/10.1016/j.esmoop.2026.106062. The OS maturity, at the time of primary publication, ranged from 12.4% to 59.5% (Table 1). The OS information fraction, where it could be determined, ranged from 24.0% to 157.0%. PFS2 maturity ranged from 27.0% to 75.4%. Long-term follow-up data were available for 10 out of 17 trials (59%). Long-term OS maturity, calculated from updated data, ranged from 45.0% to 75.4%. At any time during follow-up, exposure of the control arm to a therapeutic agent of the intervention group occurred in 0%-48.0% of patients (Supplementary Table S3, available at https://doi.org/10.1016/j.esmoop.2026.106062).

A total of 11 trials provided data on both median PFS2 and median OS (Supplementary Table S4, available at https://doi.org/10.1016/j.esmoop.2026.106062). In the intervention arms, the median PFS2/OS ratio was 0.60, with values ranging from 0.37 to 0.91. In the control arms, the median PFS2/OS ratio was 0.53, with values ranging from 0.47 to 0.79. Median PFS2 was reached in the earlier half of the OS timeline (PFS2/OS ratio <0.50) in only two trials in the intervention arms and two trials in the control arms.

### Correlation of PFS2 and OS

**Figure 2:**
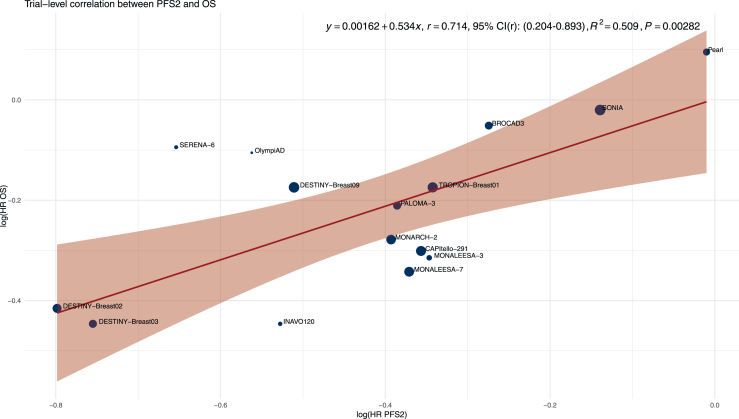
**Trial-level correlation between progression-free survival 2 (PFS2) and overall survival (OS).** Scatterplot of log(HR OS) versus log(HR PFS2) across randomized controlled trials. Point size is proportional to total sample size. The red line represents the sample size-weighted linear regression fit, with the shaded area indicating the 95% confidence interval (CI). The equation, Pearson correlation coefficient (*r*) with 95% bootstrap CI, *R*^2^, and *P* value for the regression slope are annotated. HR, hazard ratio.

A total of 15 RCTs were included in the primary trial-level surrogacy analysis of PFS2 and OS (Figure 2). The sample size-weighted Pearson correlation coefficient (*r*) between log(HR PFS2) and log(HR OS) was 0.714 (95% CI 0.204-0.893). The coefficient of determination (*R*^2^) was 0.509, indicating that ∼51% of the variability in treatment effect on OS was explained by the effect on PFS2. The corresponding linear regression yielded a slope of 0.534 (*P* = 0.003).

The correlation between PFS2 at initial reporting and OS from long-term follow-up was evaluated as a validation analysis (Supplementary Figure S1, available at https://doi.org/10.1016/j.esmoop.2026.106062). A total of nine RCTs were included. The sample size-weighted *r* was 0.552 (95% CI –0.12 to 0.897). The *R*^2^ was 0.305 (slope = 0.396, *P* = 0.123).

### PFS2 maturity

**Figure 3:**
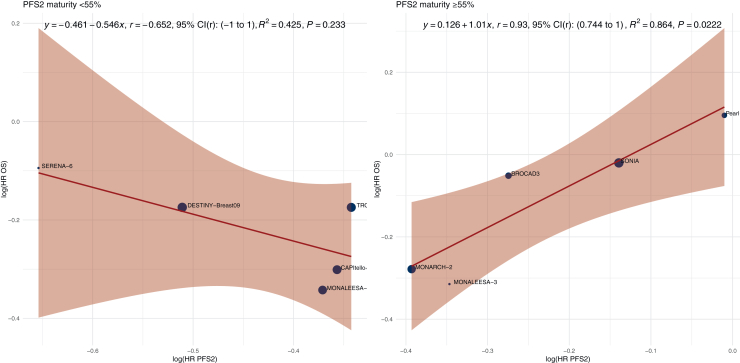
**Trial-level correlation between PFS2 and OS, stratified by PFS2 maturity.** Scatterplots of log(HR OS) versus log(HR PFS2) across randomized controlled trials grouped by PFS2 maturity (<55% versus ≥55%). Point size is proportional to total sample size. The red line represents the sample size-weighted linear regression fit, with the shaded area indicating the 95% confidence interval (CI). The equation, Pearson correlation coefficient (*r*) with 95% bootstrap CI, *R*^2^, and *P* value for the regression slope are annotated. HR, hazard ratio; OS, overall survival; PFS2, progression-free survival 2.

To assess whether surrogate validity varied according to the maturity of PFS2 data, a subgroup analysis was carried out by stratifying trials based on the proportion of patients who had experienced a PFS2 event at the time of reporting (<55% versus ≥55%) (Figure 3). The subgroup of PFS2 maturity ≥55% included five RCTs. The sample size-weighted *r* was 0.93 (95% CI 0.744-1). The *R*^2^ was 0.864 (slope = 1.01, *P* = 0.022). The subgroup of PFS2 maturity <55% included five trials. The sample size-weighted *r* was –0.652 (95% CI –1 to 1). The *R*^2^ was 0.425 (slope = –0.546, *P* = 0.233).

To validate the surrogacy based on PFS2 maturity, a secondary analysis was carried out including only trials with PFS2 maturity >55% and OS from long-term follow-up (Supplementary Figure S2, available at https://doi.org/10.1016/j.esmoop.2026.106062). A total of three RCTs were included. The sample size-weighted *r* was 0.922 (95% CI –1 to 1). The *R*^2^ was 0.85 (slope = 3.06, *P* = 0.253).

### OS information fraction

**Figure 4:**
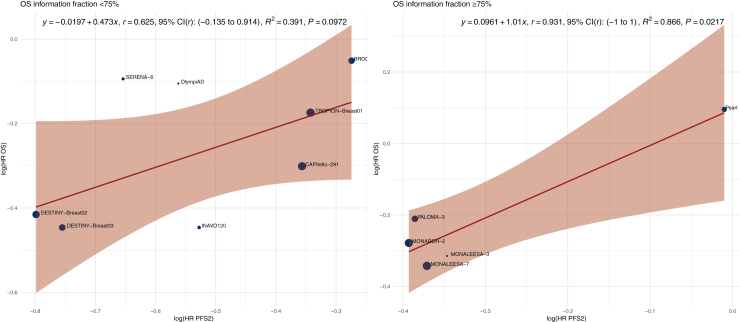
**Trial-level correlation between PFS2 and OS, stratified by OS information fraction.** Scatterplots of log(HR OS) versus log(HR PFS2) across randomized controlled trials grouped by OS information fraction (<75% versus ≥75%). Point size is proportional to total sample size. The red line represents the sample size-weighted linear regression fit, with the shaded area indicating the 95% confidence interval (CI). The equation, Pearson correlation coefficient (*r*) with 95% bootstrap CI, *R*^2^, and *P* value for the regression slope are annotated. HR, hazard ratio; OS, overall survival; PFS2, progression-free survival 2.

To assess whether surrogate validity varied according to the completeness of event accumulation for OS, a subgroup analysis was conducted based on the OS information fraction, defined as the proportion of planned OS events that had occurred at the time of analysis (<75% versus ≥75%) (Figure 4). The subgroup of OS information fraction ≥75% included five RCTs. The sample size-weighted *r* was 0.931 (95% CI –1 to 1). The *R*^2^ was 0.866 (slope = 1.01, *P* = 0.022). The subgroup of OS information fraction <75% included eight trials. The sample size-weighted *r* was 0.625 (95% CI –0.135 to 0.914). The *R*^2^ was 0.391 (slope = 0.473, *P* = 0.097).

As a validation step, we assessed the correlation between PFS2 and long-term OS in studies stratified by OS information fraction (Supplementary Figure S3, available at https://doi.org/10.1016/j.esmoop.2026.106062). Among trials with an OS information fraction ≥75% (*n* = 3), the sample size-weighted *r* was –0.813 (95% CI –1 to –0.813). The *R*^2^ was 0.662 (slope = –1.78, *P* = 0.395). Among studies with OS information fraction <75% (*n* = 4), the sample size-weighted *r* was 0.66 (95% CI –1 to 1), with an *R*^2^ of 0.436 (slope = 0.452, *P* = 0.34).

### OS maturity

To assess whether surrogate validity varied according to the maturity of OS data, a subgroup analysis was conducted based on OS maturity, defined as the proportion of planned OS events observed at the time of analysis (<40% versus 40%-60%) (Supplementary Figure S4, available at https://doi.org/10.1016/j.esmoop.2026.106062). The <40% OS maturity subgroup included nine RCTs. The sample size-weighted *r* was 0.677(95% CI –0.089 to 0.936). The *R*^2^ was 0.458 (slope = 0.473, *P* = 0.045). The 40%-60% OS maturity subgroup included six trials. The sample size-weighted *r* was 0.702 (95% CI –0.94 to 0.988). The *R*^2^ was 0.493 (slope = 0.634, *P* = 0.12).

As a validation step, we assessed the correlation between PFS2 and long-term OS in studies stratified by OS maturity (Supplementary Figure S5, available at https://doi.org/10.1016/j.esmoop.2026.106062). Among trials with OS maturity <40% (*n* = 3), the sample size-weighted *r* was 0.351 (95% CI –1 to 1). The *R*^2^ was 0.123 (slope = 0.0992, *P* = 0.772). Among studies with OS maturity between 40% and 60% (*n* = 6), the sample size-weighted *r* was 0.224 (95% CI –1 to 1), with an *R*^2^ of 0.05 (slope = 0.209, *P* = 0.718).

### Line of treatment

To examine whether the validity of PFS2 as a surrogate for OS depends on the clinical setting it reflects, a subgroup analysis was carried out based on whether PFS2 captured progression during second-line therapy versus progression on third-line or later treatment (Supplementary Figure S6, available at https://doi.org/10.1016/j.esmoop.2026.106062). Among trials in which PFS2 represented progression in second-line treatment (*n* = 4), the sample size-weighted *r* was 0.719 (95% CI –1 to 1). The *R*^2^ was 0.517 (slope = 0.63, *P* = 0.281). Among trials where PFS2 reflected progression beyond second-line therapy (*n* = 11), the *r* was 0.706 (95% CI 0.0138-0.922), with an *R*^2^ of 0.498 (slope = 0.506, *P* = 0.0152).

### Control group exposure to intervention treatment

To explore whether control group exposure to experimental group treatment influenced the surrogacy between PFS2 and OS, a subgroup analysis was conducted using long-term OS data, stratified by the proportion of control group patients who received an experimental arm agent at any point during long-term follow-up (Supplementary Figure S7, available at https://doi.org/10.1016/j.esmoop.2026.106062). Among trials with exposure rates of 0%-30% (*n* = 5), the sample size-weighted *r* was 0.429 (95% CI –1 to 1). The *R*^2^ was 0.184 (slope = 0.541, *P* = 0.471). Among trials with exposure rates between 30% and 50% (*n* = 4), the sample size-weighted *r* was 0.769 (95% CI –1 to 1), with an *R*^2^ of 0.592 (slope = 0.362, *P* = 0.231).

### PFS2 and change of OS over time

To explore whether PFS2 was related to changes in OS over time, an exploratory analysis was conducted comparing PFS2 with the difference in log(HR OS) between early and long-term OS analyses [Δlog(HR OS)] across studies reporting both time points (*n* = 8). A moderate negative correlation was observed (Spearman *ρ* = –0.505, *P* = 0.248), suggesting that trials with greater PFS2 benefit may have experienced smaller changes in OS estimates with longer follow-up, although this trend did not reach statistical significance (Supplementary Figure S8, available at https://doi.org/10.1016/j.esmoop.2026.106062).

### Correlation of median PFS2 and OS

To evaluate the association between PFS2 and OS at the arm level, a pooled analysis was conducted across treatment arms with both median PFS2 and median OS data available in months (*n* = 20). A strong correlation was observed between median PFS2 and median OS across treatment arms, with a Spearman correlation coefficient of *ρ* = 0.914 (*P* < 0.001) (Supplementary Figure S9, available at https://doi.org/10.1016/j.esmoop.2026.106062).

### Sensitivity analysis

A leave-one-out sensitivity analysis was carried out to assess the robustness of the correlation between log(HR PFS2) and log(HR OS) in the primary analysis (Supplementary Table S5, available at https://doi.org/10.1016/j.esmoop.2026.106062). When each trial was excluded in turn, the Pearson correlation coefficient (*r*) ranged from 0.511 to 0.762. The *R*^2^ values ranged from 0.390 to 0.616, and the slope estimates ranged from 0.461 to 0.596. Across all iterations, the association remained positive, with *P* values ranging from 0.0009 to 0.0122, indicating that the correlation between PFS2 and OS remained statistically significant and directionally consistent regardless of which individual trial was excluded.

### Correlation of PFS2 and OS in breast cancer subtypes

To assess whether the surrogacy relationship between PFS2 and OS differed by breast cancer subtype, we evaluated the association separately in hormone receptor-positive HER2-negative and HER2-positive disease. Analyses for triple-negative breast cancer were not feasible because of insufficient PFS2 data. A total of nine RCTs involving patients with hormone receptor-positive HER2-negative breast cancer treated with endocrine therapy were included in this analysis (Supplementary Figure S10, available at https://doi.org/10.1016/j.esmoop.2026.106062). The sample size-weighted *r* was 0.716 (95% CI –0.758 to 0.991) and the *R*^2^ was 0.512 (slope = 0.691, *P* = 0.03). Only three trials provided PFS2 data on HER2-positive disease (Supplementary Figure S11, available at https://doi.org/10.1016/j.esmoop.2026.106062). The sample size-weighted *r* was 0.976 (95% CI –1 to 1) and the *R*^2^ was 0.95 (slope = 0.928, *P* = 0.141).

### Correlation of PFS1 and OS

To provide a more comprehensive evaluation of progression-based endpoints as potential surrogates for OS, the correlation between PFS1 and OS was also explored. A total of 14 RCTs provided data on both PFS1 and OS (Supplementary Table S2, available at https://doi.org/10.1016/j.esmoop.2026.106062). The sample size-weighted Pearson correlation coefficient (*r*) between log(HR PFS1) and log(HR OS) was 0.774 (95% CI 0.409-0.949). The coefficient of determination (*R*^2^) was 0.599, indicating that ∼60% of the variability in treatment effect on OS was explained by the effect on PFS1 (Supplementary Figure S12, available at https://doi.org/10.1016/j.esmoop.2026.106062). The corresponding linear regression yielded a slope of 0.455 (*P* = 0.001).

The correlation between PFS1 at initial reporting and OS from long-term follow-up was also evaluated as a validation analysis (Supplementary Figure S13, available at https://doi.org/10.1016/j.esmoop.2026.106062). A total of eight RCTs were included. The sample size-weighted *r* was 0.747 (95% CI 0.517-0.982). The *R*^2^ was 0.56 (slope = 0.306, *P* = 0.033).

## Discussion

A recently published study demonstrated that objective response rate and PFS are poor trial-level surrogates for OS across several cancer types, particularly in breast cancer.^16^ This finding underscores the need for more robust surrogate endpoints. To our knowledge, the present study is the first to systematically evaluate PFS2 as a surrogate for OS in RCTs of breast cancer.

In our study, PFS2 demonstrated a strong correlation with OS (*r* = 0.714, 95% CI 0.204-0.893). These results are consistent with previously published findings.^5^^,^^9^ In an analysis of 38 trials across eight tumor types, five of which involved breast cancer, Woodford et al. reported a similar moderate correlation between PFS2 and OS (*r* = 0.67, 95% CI 0.08-0.69).^9^ In both this study and an updated analysis involving 42 analysis units (*r* = 0.70), the authors emphasized that PFS2 was more strongly correlated with OS than PFS1 or objective response rate, and proposed PFS2 as a suitable primary endpoint in future trials.^5^^,^^9^ However, reliance on Pearson’s *r* alone has been questioned, as it can be misleadingly high.^46^ On the other hand, the coefficient of determination (*R*^2^) offers a more interpretable estimate of how much of the variance in OS is explained by the surrogate.^46^ In our study, despite the strong correlation, PFS2 explained only 51% of the variance in OS (*R*^2^ = 0.509), suggesting that additional factors may influence this association and warrant further investigation. Notably, this relationship was attenuated in the validation analysis using long-term OS data, where the correlation with PFS2 was more modest (*r* = 0.552, *R*^2^ = 0.305). In contrast, in our analysis PFS1 demonstrated a stronger association with OS (*r* = 0.774, *R*^2^ = 0.599), explaining ∼10% more of the variance in OS compared with PFS2. This association was largely retained in the long-term OS validation analysis (*r* = 0.747, *R*^2^ = 0.56).

Several organizations have proposed frameworks to assess surrogate endpoint validity. According to the Institute for Quality and Efficiency in Health Care (IQWiG) guidelines, a surrogate endpoint is considered valid if the lower bound of the 95% CI for *r* is ≥0.85, and invalid if the upper bound is ≤0.70; values between these thresholds are deemed inconclusive.^47^ In our analysis, the correlation between PFS2 and OS yielded a lower CI bound of 0.204 and an upper bound of 0.893, classifying the surrogate validity of PFS2 as inconclusive under the IQWiG criteria. Combined with a suboptimal *R*^2^ value (0.509), this prompted us to explore whether surrogate validity improved under specific conditions, including thresholds for PFS2 maturity, OS information fraction, and OS maturity. When stratified by PFS2 maturity, studies with ≥55% maturity at the time of primary analysis explained 86% of the variation in OS, compared with 43% for studies with less mature PFS2 data. The corresponding sample size-weighted correlation coefficient for the ≥55% subgroup was *r* = 0.93 (95% CI 0.744-1), approaching thresholds proposed in regulatory guidelines for establishing a valid surrogate endpoint. Similarly, when stratifying by OS information fraction, studies with ≥75% information fraction showed an *R*^2^ of 0.866, compared with 0.391 for those with lower values. However, the regression slope in this subgroup was negative in the long-term validation analysis, raising concerns about the consistency of the association over time. Given the limited number of trials included in this analysis, it remains unclear whether this finding reflects random variation or a true attenuation of the surrogate relationship. In contrast, PFS2 maturity ≥55% consistently yielded strong explanatory power in both primary and long-term analyses, supporting its use as a more reliable stratification criterion at this stage.

Unexpected findings should be addressed. While it would be reasonable to expect that more mature OS data would yield stronger associations, our analysis showed that studies with OS maturity <40% and 40%-60% had similar explanatory power (*r* = 0.677 and 0.702, respectively). These results indicate that, within our dataset, PFS2 maturity and OS information fraction were more predictive of surrogate validity than OS maturity. Furthermore, in an exploratory analysis of change in OS benefit over time, we observed a moderate negative correlation between early PFS2 effects and the change in OS HRs from early to final data cut-offs (Spearman’s *ρ* = –0.505, *P* = 0.248). While not statistically significant, this trend may indicate that trials demonstrating large early PFS2 effects do not necessarily sustain proportional OS benefits with extended follow-up. In fact, the negative slope suggests a potential attenuation, or even reversal, of the observed OS advantage over time in some studies. Finally, another unanticipated observation concerned the proportion of patients in the control arm who were exposed to the investigational treatment. Conceptually, higher exposure rates would be expected to attenuate the observed OS benefit and thereby weaken the association between PFS2 and OS.^48^^,^^49^ However, this was not clearly supported by our exploratory subgroup analysis. Paradoxically, the correlation appeared more pronounced in trials with exposure rates of 30%-50% compared with those with lower exposure rates, although the number of studies was limited and the CIs were wide. While this counterintuitive trend should be interpreted with caution, it raises the possibility that the timing, nature, or therapeutic impact of exposure to investigational agents may differ meaningfully across trials, and that these differences are not fully captured by the percentage alone. This finding further underscores the methodological challenges of evaluating surrogate endpoints in the context of heterogeneous post-progression treatment effects, particularly when cross-over is permitted in some trials but not consistently adjusted for.^49^

The definition and operationalization of PFS2 vary across clinical trials, raising concerns about its consistency as a surrogate endpoint. In the 2012 appendix to the EMA guidelines, PFS2 was defined as the time from randomization to second objective disease progression or death.^8^ In contrast, the 2019 EMA guideline describes PFS2 as the time from randomization to objective disease progression on next-line treatment or death.^7^ This discrepancy may introduce ambiguity regarding whether PFS2 should be applied exclusively in the context of second-line therapy or more broadly across any subsequent line of treatment. In our dataset, only 6 of the 18 trials evaluated PFS2 specifically in the context of second-line treatment for advanced or metastatic breast cancer. In the stratified analysis based on whether PFS2 represented progression during second-line or later-line therapy, the correlation with OS remained similar between groups (*r* = 0.719 and 0.706, respectively). These findings suggest that the relationship between PFS2 and OS is consistent, irrespective of the clinical context in which PFS2 is measured.

Among the trials included in our analysis, TANIA^44^ and SONIA^19^ were the only studies that evaluated PFS2 as a primary endpoint, and in both cases, it was aligned with second-line treatment. Specifically in the SONIA trial protocol, the authors made continuous adjustments to the definition of PFS2.^19^ The investigators accounted for complex treatment scenarios, including patients who continued first-line therapy beyond radiologic progression and those who transitioned to second-line treatment without documented first progression. In addition, they specified that initiation of chemotherapy would constitute a PFS2 event, as it signaled failure of the treatment strategy, whereas initiation of a new endocrine therapy would not trigger a PFS2 event under their definition.^19^ This level of definitional nuance highlights the potential for variability in how PFS2 is operationalized across trials, even when designated as a primary endpoint, and reinforces the need for standardized, transparent criteria to ensure consistency and comparability in surrogate endpoint evaluation.

This study has several notable strengths. To our knowledge, this is the first cancer-specific analysis to systematically evaluate PFS2 as a surrogate for OS, focusing exclusively on randomized trials in breast cancer. The use of multiple complementary statistical approaches, including both correlation and explanatory metrics, allowed for a robust assessment of surrogate validity. Subgroup analyses further explored the influence of key trial-level factors such as endpoint maturity, treatment line, and exposure to intervention group treatment. In addition, the availability of long-term OS data in a subset of trials enabled validation of early findings over time, an important and often overlooked component in surrogate endpoint research. However, certain limitations should be considered. The primary limitation of this study is the limited number of available RCTs, which constrained the precision of trial-level surrogacy estimates and resulted in wide CIs around correlation coefficients. This limited sample size also precluded extensive stratified analyses by therapeutic strategy or breast cancer subtype and reduced the statistical power of other analyses. This was particularly evident in the long-term OS analyses, where limited data may have contributed to unexpected or inverse associations. Moreover, as this was a trial-level analysis based on publicly available data, patient-level surrogacy could not be evaluated. Finally, the possibility of incomplete reporting cannot be excluded, as PFS2 data from three trials were available only from congress abstracts. Nevertheless, these data were included to maximize completeness of outcome reporting based on a rigorous and systematic literature search.

In conclusion, PFS2 demonstrates a moderate and context-dependent association with OS in breast cancer trials rather than robust trial-level surrogacy. Although correlations between PFS2 and OS were observed, PFS2 explained only a limited proportion of the variance in OS, and this association was not consistently retained in analyses using long-term survival data. PFS2 maturity and OS information fraction are critical factors that influence the strength of surrogate validity, emphasizing the need to account for these methodological factors in future evaluations. Collectively, these findings underscore the need for caution in interpreting PFS2 as a surrogate endpoint and highlight the importance of considering endpoint maturity and clinical context when evaluating its use in future trials.
