# Supplementary material for: Progression-free survival 2 (PFS2) as a surrogate endpoint for overall survival (OS) in breast cancer randomized controlled clinical trials
Source: ESMO Open. 2026 Jan 27;11(2):106062. doi: 10.1016/j.esmoop.2026.106062 (PMC12865639; doi:10.1016/j.esmoop.2026.106062)
Supplement: Supplementary Data [file mmc1.docx]

***Supplementary Material***

**Progression-free survival 2 (PFS2) as a surrogate endpoint for overall survival (OS) in breast cancer randomized controlled clinical trials**

***Supplementary Figures***


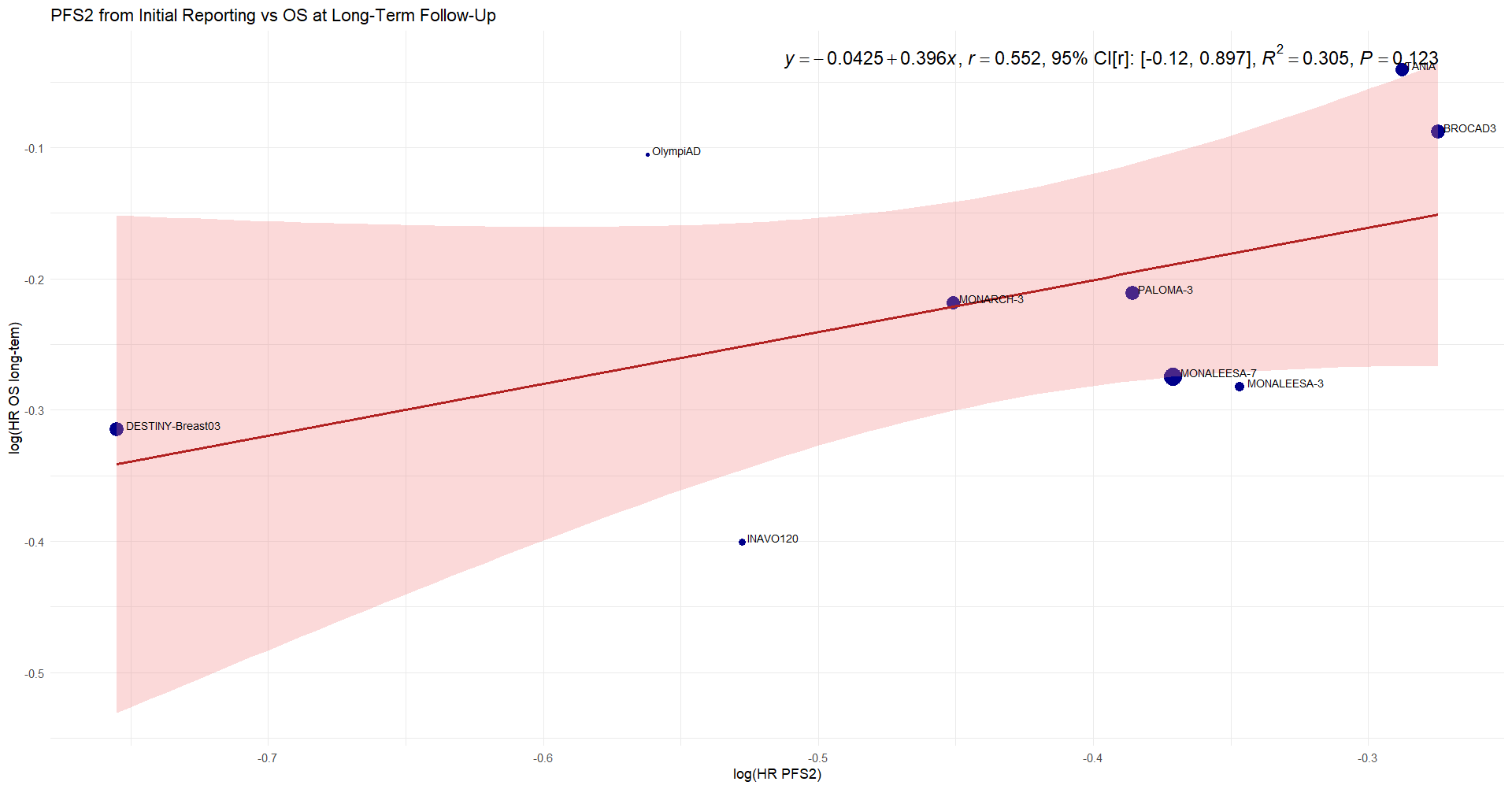


**Supplementary Figure 1**. Association between PFS2 from initial reporting and OS at long-term follow-up. Scatterplot of log(HR_OS) at long-term follow-up versus log(HR_PFS2) from the initial analysis. Point size is proportional to trial sample size. A weighted linear regression line is shown with 95% confidence interval shading. The annotated text includes the regression equation, weighted Pearson correlation coefficient (r) with 95% bootstrap confidence interval (CI), R², and regression P-value. HR, hazard ratio.


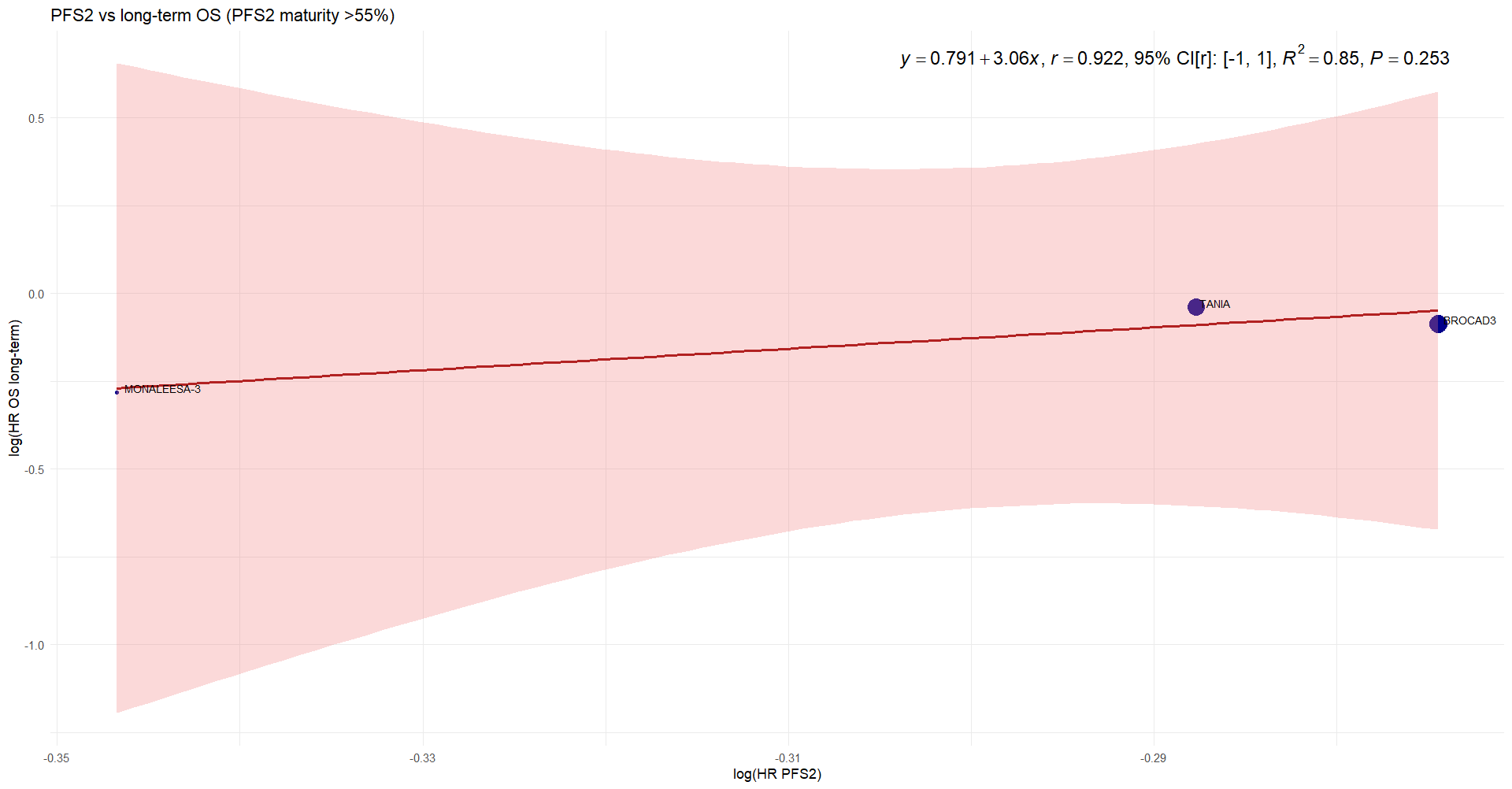


**Supplementary Figure 2**. Trial-level correlation between PFS2 from initial reporting and long-term OS in trials with PFS2 maturity >55%. Scatterplot of log(HR_OS) versus log(HR_PFS2) across randomized controlled trials with PFS2 maturity >55% and long-term OS data. Point size is proportional to total sample size. The red line represents the sample size–weighted linear regression fit, with shaded area indicating the 95% confidence interval. The equation, Pearson correlation coefficient (r) with 95% bootstrap confidence interval (CI), R², and P-value for the regression slope are annotated. HR, hazard ratio.


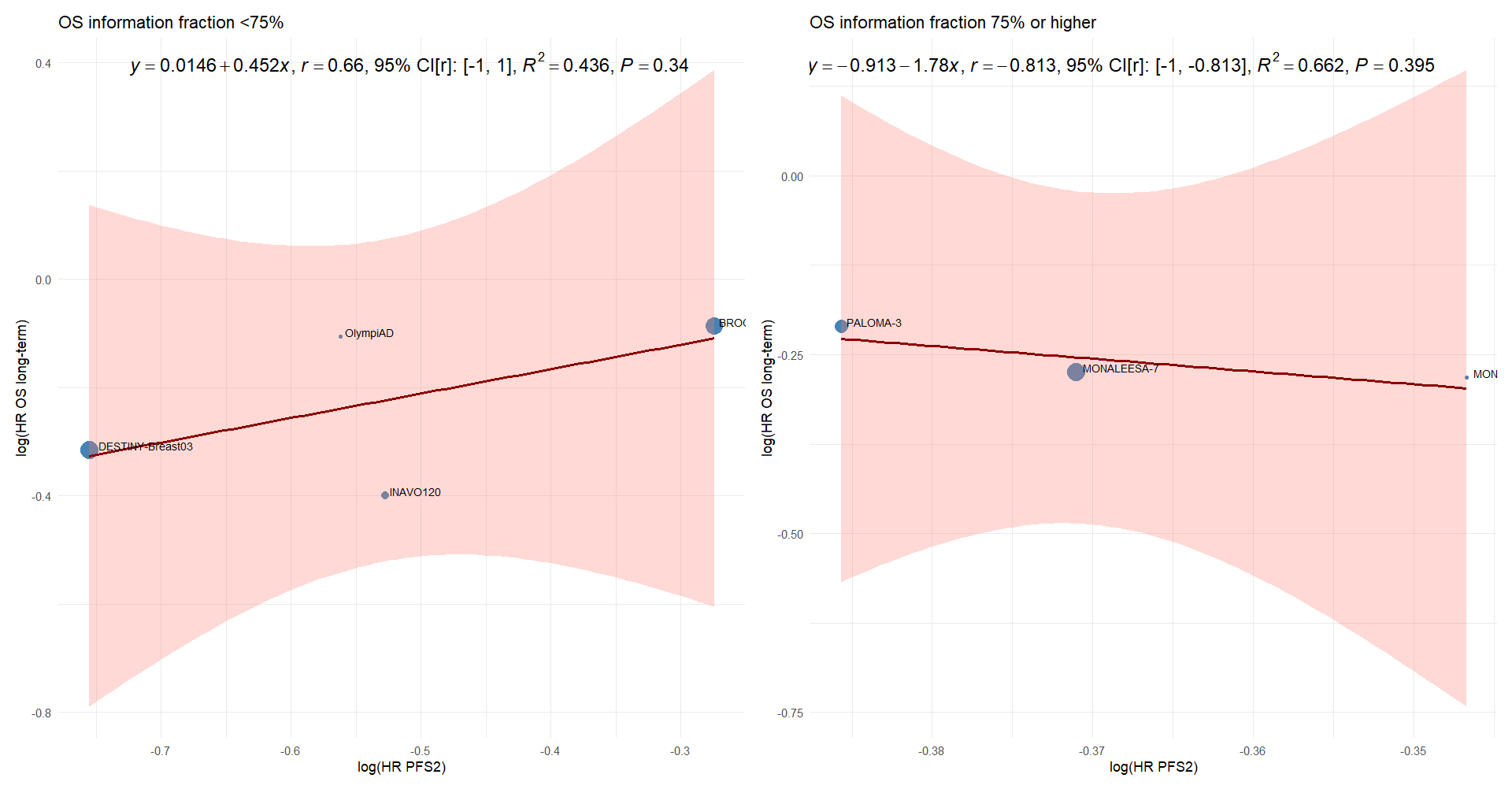


**Supplementary Figure 3**. Trial-level correlation between PFS2 from initial reporting and long-term OS, stratified by OS information fraction. Scatterplots of log(HR_OS) versus log(HR_PFS2) across randomized controlled trials grouped by OS information fraction (<75% vs ≥75%). Point size is proportional to total sample size. The red line represents the sample size–weighted linear regression fit, with shaded area indicating the 95% confidence interval. The equation, Pearson correlation coefficient (r) with 95% bootstrap confidence interval (CI), R², and P-value for the regression slope are annotated. HR, hazard ratio.


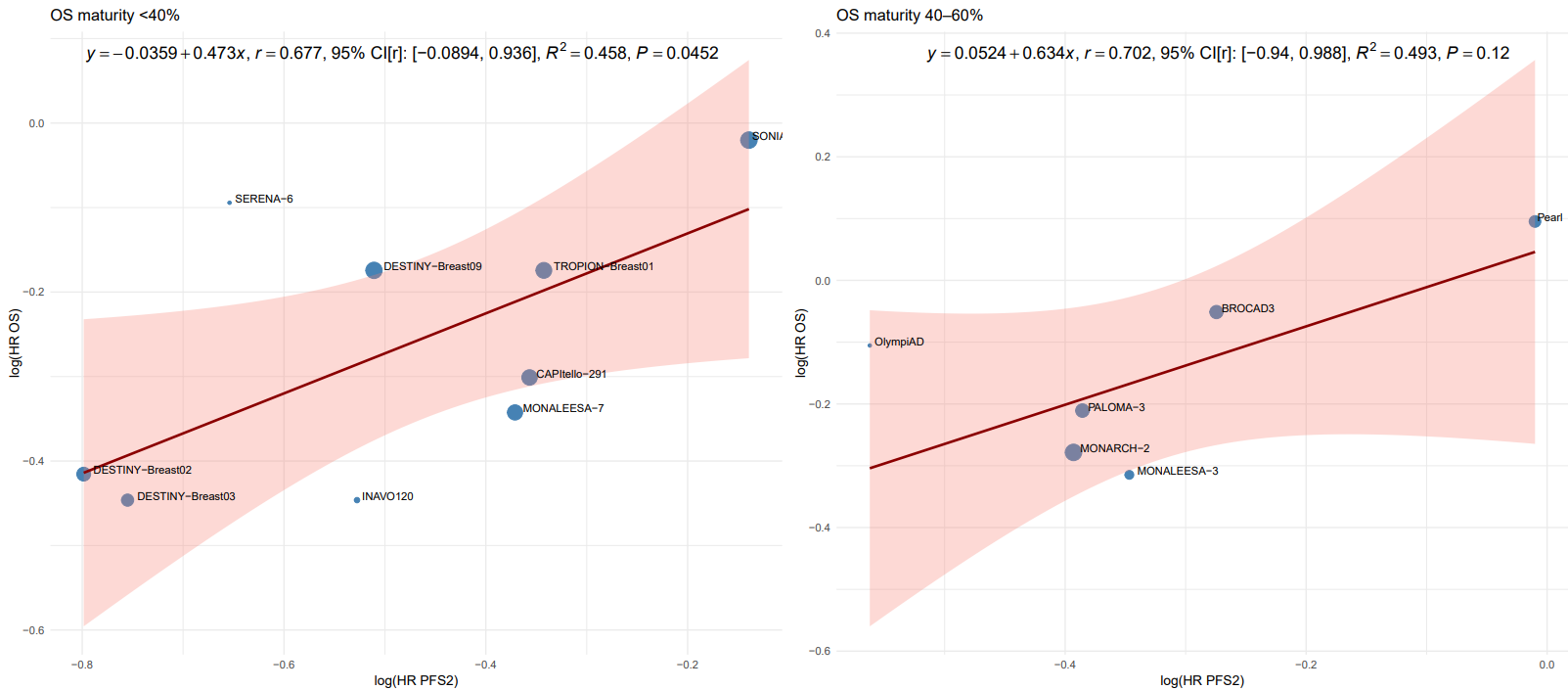


**Supplementary Figure 4.** Trial-level correlation between PFS2 and OS, stratified by OS maturity. Scatterplots of log(HR_OS) versus log(HR_PFS2) across randomized controlled trials grouped by OS maturity (<40% vs 40-60%). Point size is proportional to total sample size. The red line represents the sample size–weighted linear regression fit, with shaded area indicating the 95% confidence interval. The equation, Pearson correlation coefficient (r) with 95% bootstrap confidence interval (CI), R², and P-value for the regression slope are annotated. HR, hazard ratio.


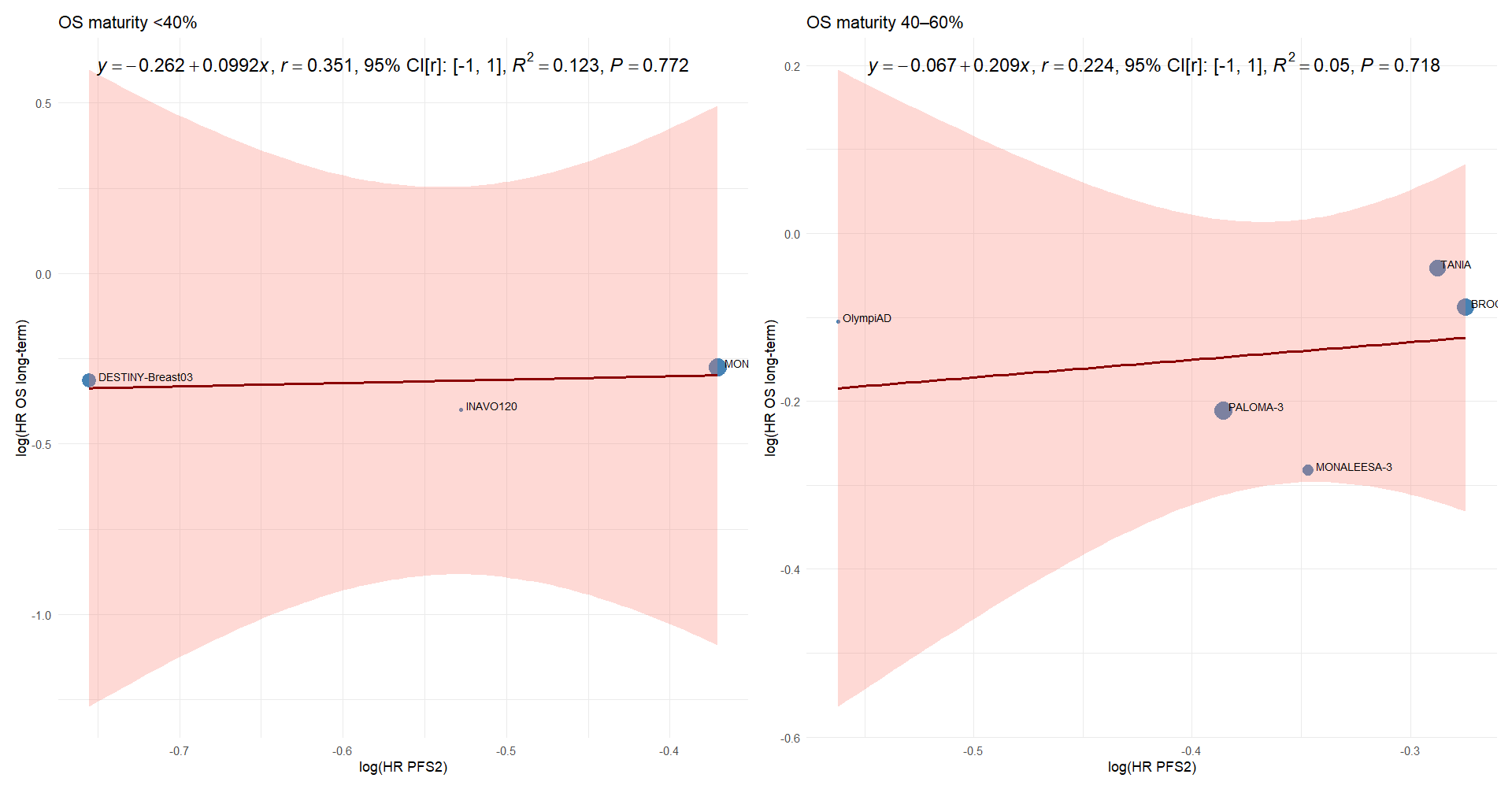


**Supplementary Figure 5**. Trial-level correlation between PFS2 from initial reporting and long-term OS, stratified by OS maturity. Scatterplots of log(HR_OS) versus log(HR_PFS2) across randomized controlled trials grouped by OS maturity (<40% vs 40–60%). Point size is proportional to total sample size. The red line represents the sample size–weighted linear regression fit, with shaded area indicating the 95% confidence interval. The equation, Pearson correlation coefficient (r) with 95% bootstrap confidence interval (CI), R², and P-value for the regression slope are annotated. HR, hazard ratio.


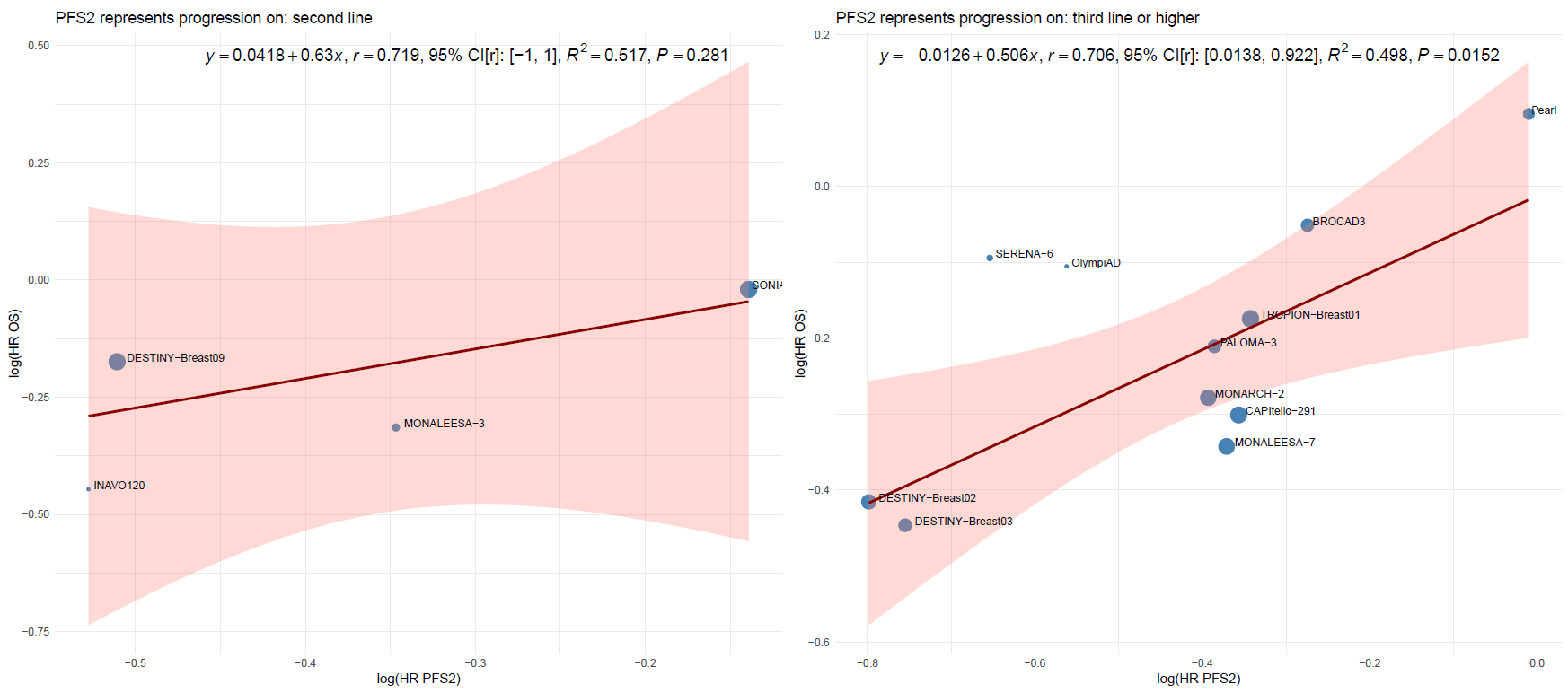


**Supplementary Figure 6**. Trial-level correlation between PFS2 and OS, stratified by the line of treatment that is represented by PFS2. Scatterplots of log(HR_OS) versus log(HR_PFS2) across randomized controlled trials in which PFS2 captured progression during second-line treatment (left) versus third-line or later treatment (right). Point size is proportional to total sample size. The red line represents the sample size–weighted linear regression fit, with shaded area indicating the 95% confidence interval. The equation, Pearson correlation coefficient (r) with 95% bootstrap confidence interval (CI), R², and P-value for the regression slope are annotated. HR, hazard ratio.


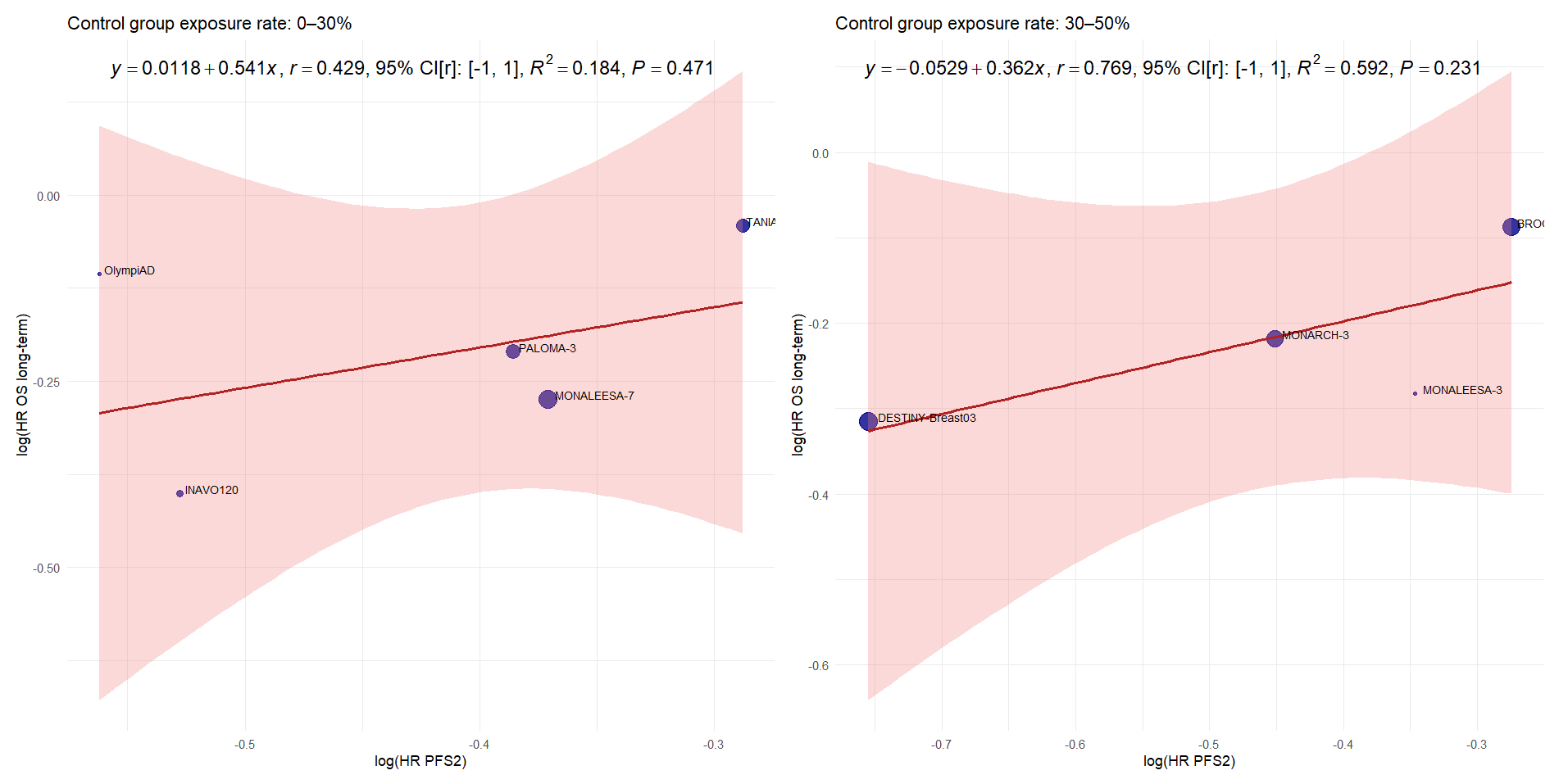


**Supplementary Figure 7**. Trial-level correlation between PFS2 and long-term OS, stratified by the percentage of the control group that was exposed to a therapeutic agent of the experimental group at long-term follow-up. Scatterplots of log(HR_OS) versus log(HR_PFS2) across randomized controlled trials with low (0–30%, left) versus moderate (30–50%, right) crossover rates. Point size is proportional to total sample size. The red line represents the sample size–weighted linear regression fit, with shaded area indicating the 95% confidence interval. The equation, Pearson correlation coefficient (r) with 95% bootstrap confidence interval (CI), R², and P-value for the regression slope are annotated. HR, hazard ratio.


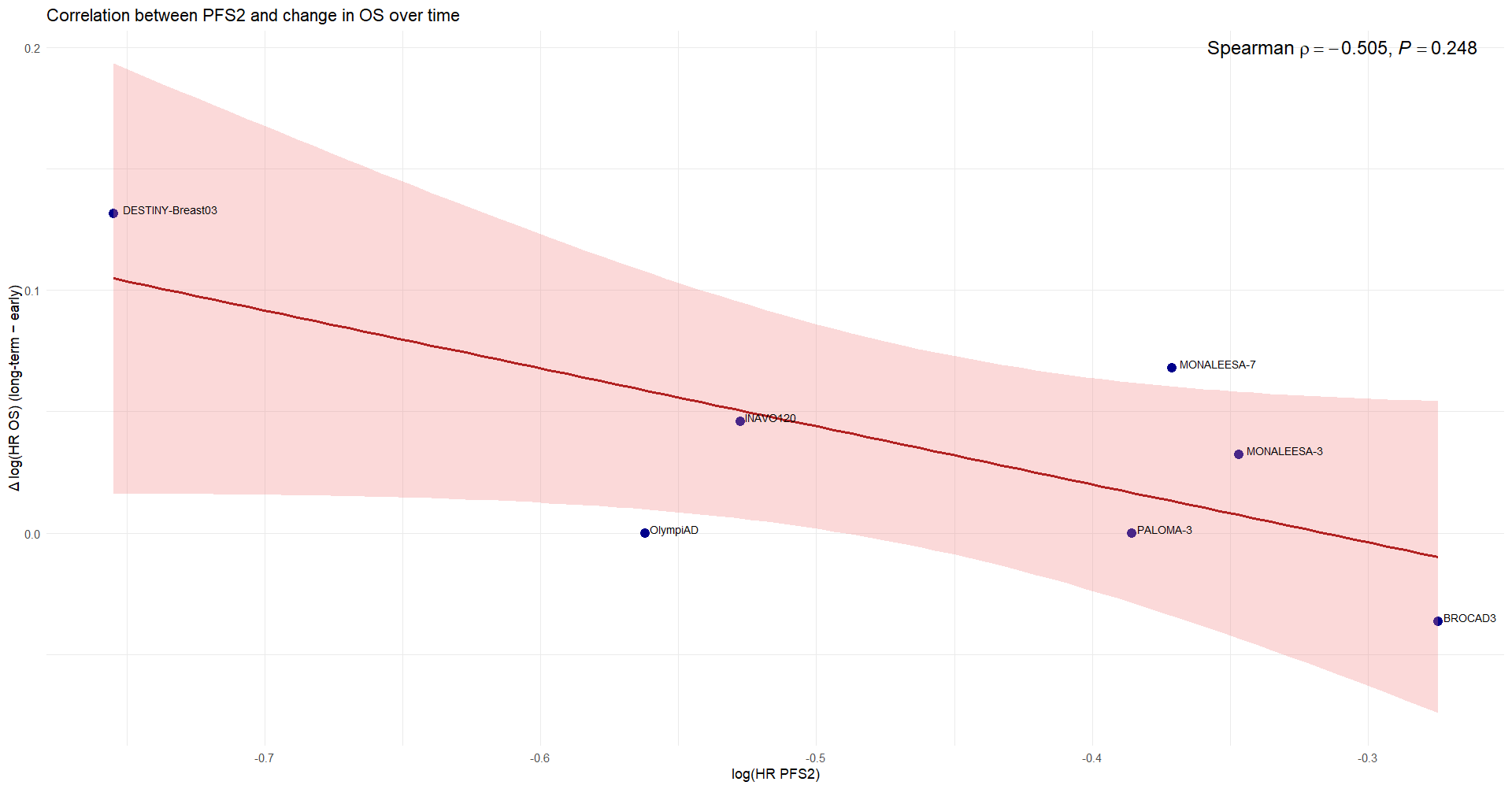


**Supplementary Figure 8**. Correlation between PFS2 and change in OS over time. Scatterplot of log(HR_PFS2) versus Δlog(HR_OS) (defined as the difference between long-term and initial OS estimates) across trials reporting both endpoints. The red line represents the linear regression fit with 95% confidence interval (shaded area). The Spearman rank correlation coefficient (ρ) and associated P-value are annotated. HR, hazard ratio.


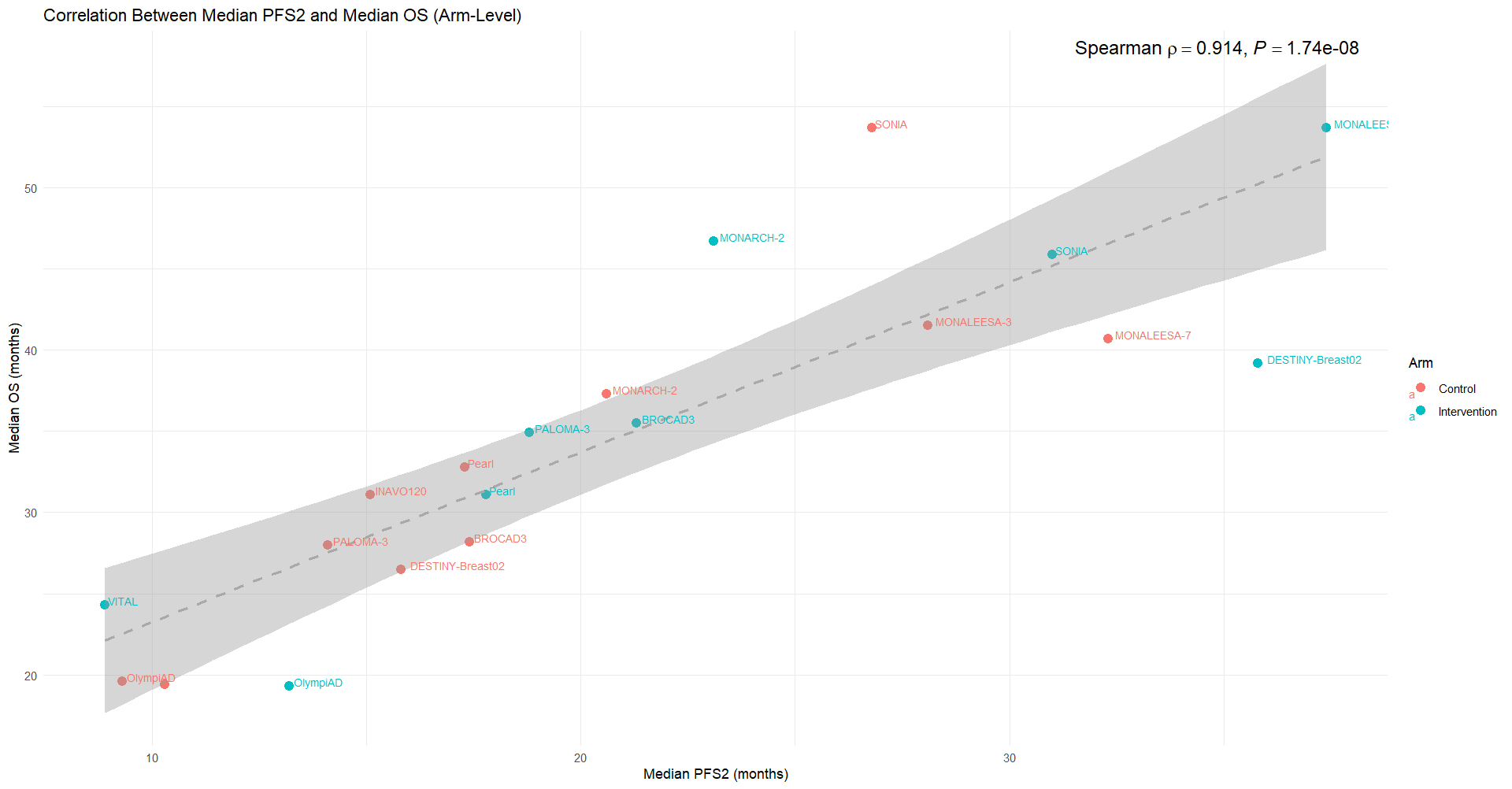


**Supplementary Figure 9**. Arm-level correlation between median PFS2 and median OS. Scatterplot of median OS versus median PFS2 (in months) for treatment arms with available data across randomized controlled trials. Points are colored by treatment assignment (intervention or control). The dashed line represents a linear regression fit with a shaded 95% confidence interval. The Spearman correlation coefficient (ρ) and associated P-value are annotated.


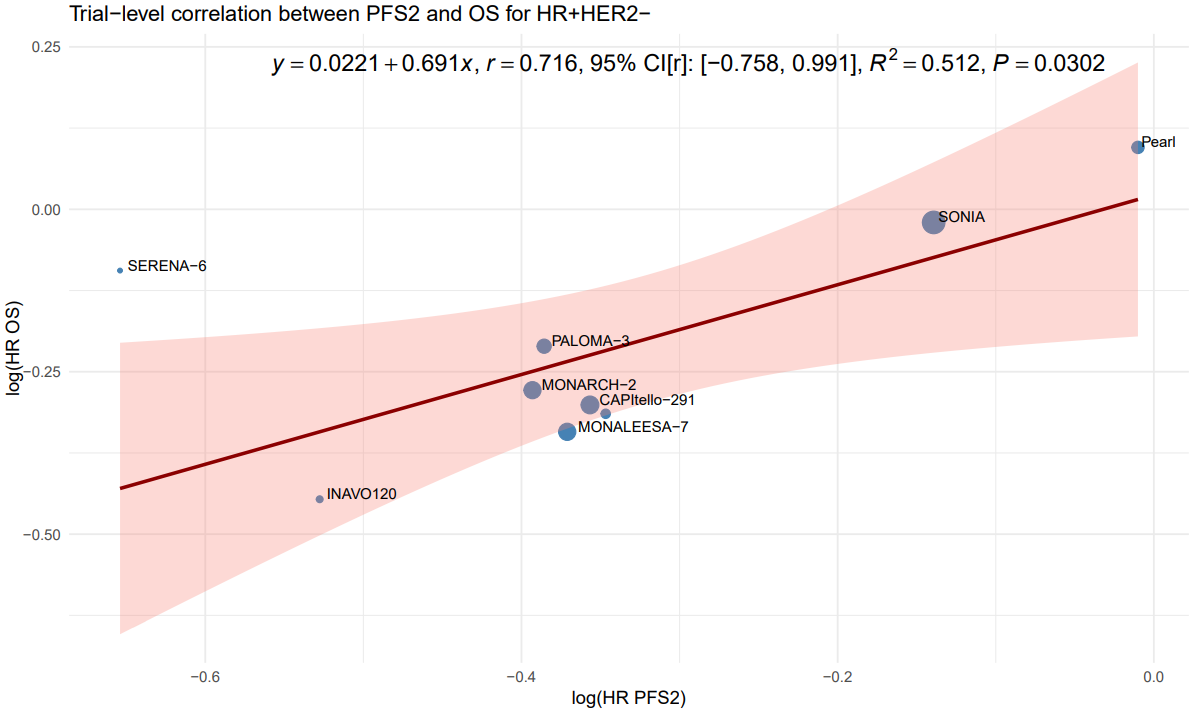


**Supplementary Figure 10**. Trial-Level correlation between PFS2 and OS for hormone receptor-positive, HER2-negative (HR+HER2-) breast cancer. Scatterplot of log(HR_OS) versus log(HR_PFS2) across randomized controlled trials. Point size is proportional to total sample size. The red line represents the sample size-weighted linear regression fit, with shaded area indicating the 95% confidence interval. The equation, Pearson correlation coefficient (r) with 95% bootstrap confidence interval (CI), R², and P-value for the regression slope are annotated. HR, hazard ratio.


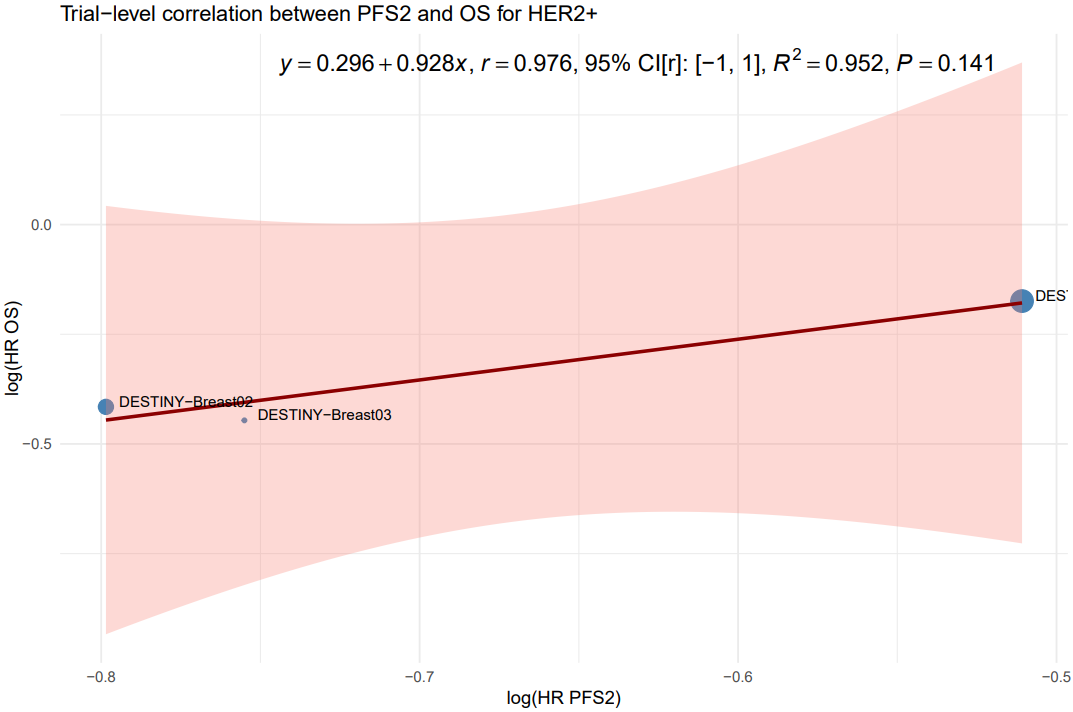


**Supplementary Figure 11**. Trial-Level correlation between PFS2 and OS for HER2-positive breast cancer. Scatterplot of log(HR_OS) versus log(HR_PFS2) across randomized controlled trials. Point size is proportional to total sample size. The red line represents the sample size-weighted linear regression fit, with shaded area indicating the 95% confidence interval. The equation, Pearson correlation coefficient (r) with 95% bootstrap confidence interval (CI), R², and P-value for the regression slope are annotated. HR, hazard ratio.


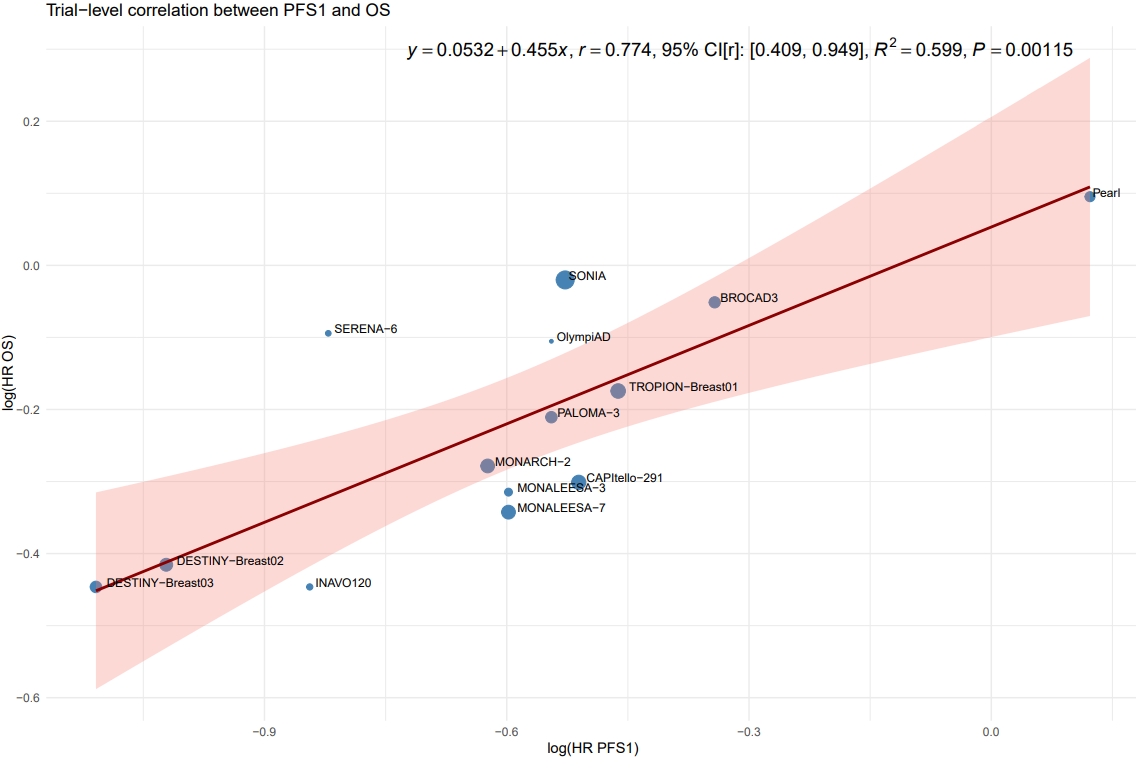


**Supplementary Figure 12**. Trial-Level correlation between progression-free survival 1 (PFS1) and OS. Scatterplot of log(HR_OS) versus log(HR_PFS1) across randomized controlled trials. Point size is proportional to total sample size. The red line represents the sample size-weighted linear regression fit, with shaded area indicating the 95% confidence interval. The equation, Pearson correlation coefficient (r) with 95% bootstrap confidence interval (CI), R², and P-value for the regression slope are annotated. HR, hazard ratio.


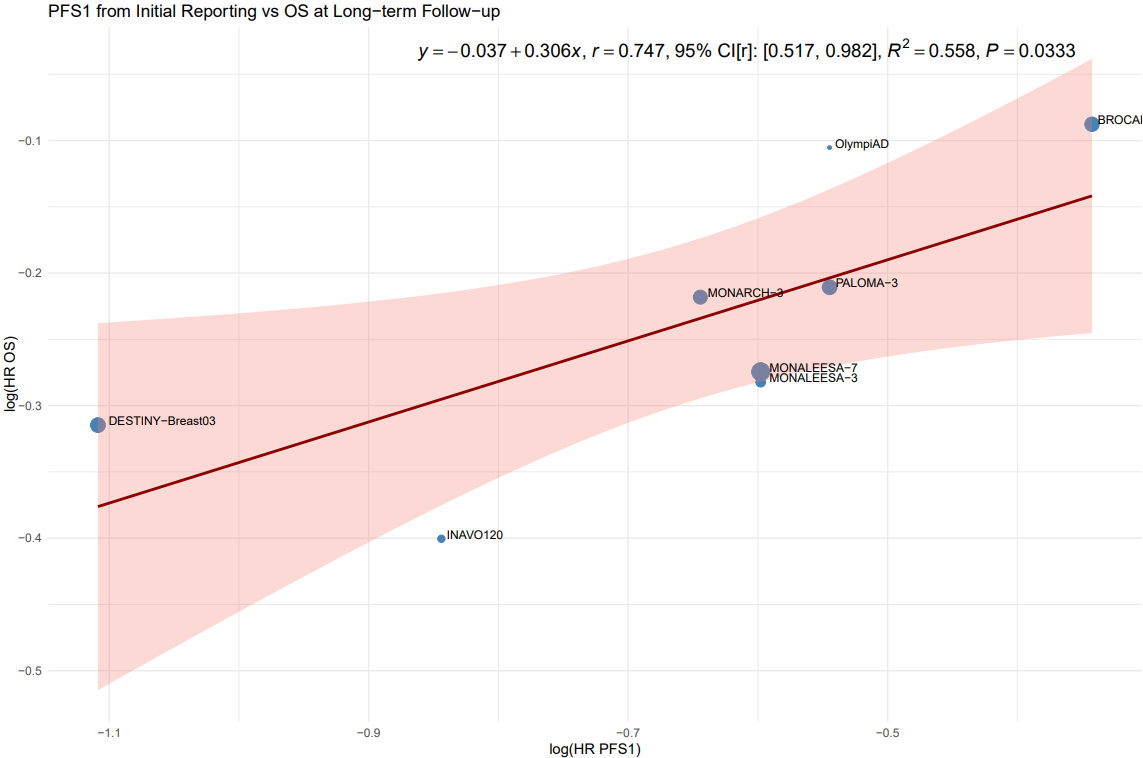


**Supplementary Figure 13**. Association between PFS1 from initial reporting and OS at long-term follow-up. Scatterplot of log(HR_OS) at long-term follow-up versus log(HR_PFS1) from the initial analysis. Point size is proportional to trial sample size. A weighted linear regression line is shown with 95% confidence interval shading. The annotated text includes the regression equation, weighted Pearson correlation coefficient (r) with 95% bootstrap confidence interval (CI), R², and regression P-value. HR, hazard ratio.

***Supplementary Tables***

| **Supplementary Table 1**: List of excluded studies with reason for exclusion | |
| --- | --- |
| PMID | Reason for exclusion |
| 39822368 | Retrospective cohort |
| 38574190 | Retrospective cohort |
| 34176251 | Retrospective cohort |
| 33302999 | Retrospective cohort |
| 32923865 | Retrospective cohort |
| 31330065 | Retrospective cohort |
| 18952558 | Retrospective cohort |
| 38417742 | Prospective cohort |
| 39271844 | Prospective cohort |
| 38414929 | Prospective cohort |
| 16614227 | Prospective cohort |
| 15111763 | Prospective cohort |
| 27315546 | Non-comparative trial |

| **Supplementary Table 3:** Detailed characteristics of the studies that provided published data on long-term follow-up | | | | | | | | | | | |
| --- | --- | --- | --- | --- | --- | --- | --- | --- | --- | --- | --- |
| **PMID** | **Author** | **Year** | **Median follow-up (months)** | **Long-term OS maturity (%)** | **Control exposure to experimental therapy (%)** | **Hazard ratio (95%CI) PFS** | **Median PFS2 intervention** | **Median PFS2 control** | **Hazard ratio (95%CI) OS** | **Median OS intervention** | **Median OS control** |
| 40454641 | Jhaveri | 2025 | 34,2 | 47,4 | 15,5 | 0,51 (0,38-0,68) | 24 | 14,5 | 0,67 (0,48-0,94) | 34 | 27 |
| 38825627 | Cortes | 2024 | 43 | 45 | 32,3 | 0,53 (0,41-0,68) | 45,2 | 23,1 | 0,73 (0,56-0,94) | 52,6 | 42,7 |
| 34965945 | Lu | 2022 | 53,5 | 45,8 | 26 | 0,68 (0,56-0,83) | 44,2 | 31 | 0,76 (0,61-0,96) | 58,7 | 48 |
| 37653397 | Neven | 2023 | 70,8 | 59,9 | 35 | 0,64 (0,49-0,84) | 50,7 | 34,6 | 0,754 (0,62-0,916) | 52,2 | 41,5 |
| 38729566 | Goetz | 2024 | 97,2 | 63,7 | 31,5 |  |  |  | 0,804 (0,637-1,015) | 66,8 | 53,7 |
| 38309017 | Dieras | 2024 | 65,7 | 70,3 | 48 |  |  |  | 0,916 (0,736-1,14) | 32,4 | 28,2 |
| 35552673 | Cristofanilli | 2022 | 73,3 | 75,4 | 22,2 |  |  |  | 0,81 (0,65-0,99) | 34,8 | 28 |
| 30689707 | Robson | 2019 | 25,3 | 63,6 | 8,2 |  |  |  | 0,9 (0,66-1,23) | 19,3 | 17,1 |
| 27502725 | Vrdoljak | 2016 | 32,1 | 64,6 | 0 | 0,79 | 6,3 | 4,2 | 0,96 (0,76-1,21) | 19,7 | 18,7 |
| 26384789 | Janni | 2015 | 18,9 | 58,9 | 35,1 |  |  |  |  | 23,3 | 20,3 |

| **Supplementary Table 4**: Median PFS2 and median OS (in months) across trials, as well as the PFS2/OS ratio. | | | | | | | | | |
| --- | --- | --- | --- | --- | --- | --- | --- | --- | --- |
| PMID | Trial name | N intervention | N control | Median PFS2 (Intervention) | Median OS (Intervention) | Median PFS2 (Control) | Median OS (Control) | PFS2/OS ratio (Intervention) | PFS2/OS ratio (Control) |
| 39604725 | SONIA | 524 | 526 | 31 | 45.9 | 26.8 | 53.7 | 0.68 | 0.5 |
| 39476340 | INAVO120 | 161 | 164 | 24 | Not reported | 15.1 | 31.1 | Not applicable | 0.49 |
| 37086745 | DESTINY-Breast02 | 406 | 202 | 35.8 | 39.2 | 15.8 | 26.5 | 0.91 | 0.6 |
| 35429901 | Pearl | 149 | 299 | 17.8 | 31.1 | 17.3 | 32.8 | 0.57 | 0.53 |
| 31166679 | MONALEESA-7 | 335 | 337 | Not reported | Not reported | 32.3 | 40.7 | Not applicable | 0.79 |
| 34102253 | MONALEESA-3 | 237 | 128 | 37.4 | 53.7 | 28.1 | 41.5 | 0.7 | 0.68 |
| 31563959 | MONARCH-2 | 446 | 223 | 23.1 | 46.7 | 20.6 | 37.3 | 0.49 | 0.55 |
| 32861273 | BROCAD3 | 337 | 172 | 21.3 | 35.5 | 17.4 | 28.2 | 0.6 | 0.62 |
| 30345905 | PALOMA-3 | 347 | 174 | 18.8 | 34.9 | 14.1 | 28 | 0.54 | 0.5 |
| 28578601 | OlympiAD | 205 | 97 | 13.2 | 19.3 | 9.3 | 19.6 | 0.68 | 0.47 |
| 24402830 | VITAL | 75 | 37 | 8.9 | 24.3 | 10.3 | 19.4 | 0.37 | 0.53 |

| **Supplementary Table 5:** Leave-one-out sensitivity analysis for the primary analysis of PFS2 and OS correlation | | | | | | |
| --- | --- | --- | --- | --- | --- | --- |
| **Excluded Trial** | **Slope** | **R_squared** | **Pearson_r** | **CI_r_lower** | **CI_r_upper** | **P_value** |
| SERENA-6 | 0,596 | 0,616 | 0,762 | 0,295 | 0,924 | 0,000885 |
| DESTINY-Breast09 | 0,558 | 0,548 | 0,665 | 0,103 | 0,916 | 0,00247 |
| TROPION-Breast01 | 0,533 | 0,505 | 0,649 | 0,031 | 0,891 | 0,00439 |
| SONIA | 0,488 | 0,42 | 0,602 | -0,104 | 0,873 | 0,0122 |
| INAVO120 | 0,515 | 0,514 | 0,658 | -0,057 | 0,916 | 0,00388 |
| CAPItello-291 | 0,549 | 0,549 | 0,674 | 0,082 | 0,917 | 0,00244 |
| DESTINY-Breast03 | 0,503 | 0,43 | 0,584 | -0,142 | 0,899 | 0,0109 |
| DESTINY-Breast02 | 0,544 | 0,434 | 0,595 | -0,144 | 0,905 | 0,0104 |
| Pearl | 0,461 | 0,39 | 0,511 | -0,13 | 0,851 | 0,017 |
| MONALEESA-7 | 0,548 | 0,568 | 0,684 | 0,146 | 0,916 | 0,00184 |
| MONALEESA-3 | 0,544 | 0,535 | 0,681 | 0,145 | 0,917 | 0,00293 |
| MONARCH-2 | 0,537 | 0,522 | 0,66 | 0,069 | 0,901 | 0,00353 |
| BROCAD3 | 0,512 | 0,494 | 0,633 | 0,019 | 0,91 | 0,00505 |
| PALOMA-3 | 0,534 | 0,509 | 0,652 | 0,099 | 0,908 | 0,00416 |
| OlympiAD | 0,561 | 0,563 | 0,708 | 0,176 | 0,927 | 0,002 |
