# Supplementary material for: Progression-free survival 2 (PFS2) as a surrogate endpoint for overall survival (OS) in breast cancer randomized controlled clinical trials
Source: ESMO Open. 2026 Jan 27;11(2):106062. doi: 10.1016/j.esmoop.2026.106062 (PMC12865639; doi:10.1016/j.esmoop.2026.106062)
Supplement: Supplementary Table 2 [file mmc2.docx]

| **Supplementary Table 2:** Details of the included studies regarding treatments, follow-up duration, and hazard ratios with corresponding 95% confidence intervals (CI) and duration of survival. | | | | | | | | | | | | | | |
| --- | --- | --- | --- | --- | --- | --- | --- | --- | --- | --- | --- | --- | --- | --- |
| **PMID** | **Trial name** | **Author** | **Year** | **N intervention** | **N control** | **Intervention treatment** | **Control treatment** | **Main regimens in next line** | **Received intervention agent in 2^nd^ line (% Control arm)** | **Median follow up (months)** | **PFS2 definition** | **Hazard ratio (95% CI) PFS1** | **Hazard ratio (95% CI) PFS2** | **Hazard ratio (95% CI) OS** |
| 40454637 | SERENA-6 | Bidard | 2025 | 157 | 158 | camizestrant+CDK4/6i+placebo | AI+CDK4/6i+placebo | ET, ADC, chemo | 34.9 | 12,6 | time from randomization to the earliest of either subsequent disease progression after the next line of therapy or death | 0,44 (0,31-0,6) | 0,52 (0,33-0,81) | 0,91 (0,48-1,73) |
| doi.org/10.1200/JCO.2025.43.17_suppl.LBA1008 | DESTINY-Breast09 | Tolaney | 2025 | 383 | 387 | T-DXd+pertuzumab | Taxane+trastuzumab+pertuzumab |  |  | 29,2 | NR |  | 0,6 (0,45 -0,79) | 0,84 (0,59-1,19) |
| 39265124 | TROPION-Breast01 | Bardia | 2025 | 365 | 367 | Dato-DXd | eribulin/vinorelbine/capecitabine/gemcitabine | ADC, ET, chemo | 4.1 | 10,8 | time to second progression or death | 0,63 (0,52-0,76) | 0,71 (0,55-0,92) | 0,84 (062-1,14) |
| 39604725 | SONIA | Sonke | 2024 | 524 | 526 | NSAI + CDK4/6i | NSAI | Fulvestrant, CDK4/6i | 72.8 | 37,3 | time from randomization until objective disease progression, symptomatic deterioration, or initiation of a new therapeutic agent on second line treatment, death, or progression during a break in initial therapy and without further therapy within one month, whichever occurs first | 0,59 (0,51-0,69) | 0,87 (0,71-1,03) | 0,98 (0,8-1,2) |
| 39476340 | INAVO120 | Turner | 2024 | 161 | 164 | inavolisib + pablociclib + fulvestrant | placebo + pablociclib + fulvestrant | chemo |  | 21,3 | Time to end of next-line treatment (proxy for time to second objective disease progression [PFS2]), defined as the time from randomization to end or discontinuation of next-line treatment, or death from any cause (whichever occurs first) | 0,43 (0,32-0,59) | 0,59 (0,42-0,83) | 0,64 (0,43-0,97) |
| 37256976 | CAPItello-291 | Turner | 2023 | 355 | 353 | capivasertib + fulvestrant | placebo + fulvestrant | HT, chemo, targeted therapy |  | 13 | time from randomisation to second progression (i.e. the earliest of either death or a progression event following treatment start after first progression) | 0,6 (0,51-0,71) | 0,7 (0,57-0,86) | 0,74 (0,56-0,98) |
| 36495879 | DESTINY-Breast03 | Hurvitz | 2023 | 261 | 263 | trastuzumab deruxtecan | trastuzumab emtasine | systemic therapy | 27 | 28,4 | time from the date of randomization to the first documented progression on the next line of therapy or death due to any cause, whichever occurred first. Thh next line of therapy was defined as the first new systemic antineoplastic therapy initiated after discontinuation of study treatment regardless of the reason for end of treatment. | 0,33 (0,26-0,43) | 0,47 (0,35-0,62) | 0,64 (0,47-0,87) |
| 37086745 | DESTINY-Breast02 | Andre | 2023 | 406 | 202 | trastuzumab deruxtecan | treatment of physician's choice | systemic therapy | 27 | 21,5 | time from randomisation to first progression on the next line of therapy or death due to any cause, whichever occurred first | 0,36 (0,28-0,45) | 0,45 (0,34-0,59) | 0,66 (0,5-0,86) |
| 35429901 | Pearl | Martin | 2022 | 149 | 299 | palbociclib + fulvestrant | capecitabine | ET, systemic therapy | 68 | 28 | time from randomization to the end of the first subsequent therapy or death from any cause | 1,13 (0,85-1,5) | 0,99 (0,76-1,29) | 1,1 (0,81-1,5) |
| 34965945 | MONALEESA-7 | Lu | 2022 | 335 | 337 | ribociclib + goserelin + NSAI/Tamoxifen | placebo + goserelin + NSAI/Tamoxifen | HT, chemo | 3.9 | 34,6 | time from randomization to the first documented disease progression while the patient was receiving second-line therapy (as reported by the physician) or death from any cause, whichever occurred first | 0,55 (0,44-0,69) | 0,69 (0,55-0,87) | 0,71 (0,54-0,95) |
| 34102253 | MONALEESA-3 | Slamon | 2021 | 237 | 128 | fulvestrant + ribociclib | fulvestrant + placebo | ET, chemo | 5 | 56,3 | the time from randomization to the frst documented disease progression while the patient was receiving next-line therapy or death from any cause, whichever occurred first | 0,55 (0,42-0,72) | 0,707 (0,57-0,84) | 0,73 (0,59-0,9) |
| 34158513 | MONARCH-3 | Jonhston | 2021 | 328 | 165 | abemaciclib + NSAI | placebo +NSAI | ET | 16.4 | 39 | time from randomization to the discontinuation date of next-line (first line of post-discontinuation therapy), or starting date of the second line of post-discontinuation therapy or death from any cause, whichever was earlier | 0,53 (0,42-0,67) | 0,637 (0,495-0,819) |  |
| 31563959 | MONARCH-2 | Sledge | 2020 | 446 | 223 | abemaciclib + fulvestrant | placebo + fulvestrant | ET, chemo, everolimus-based | 17 | 47,7 | time from randomization to discontinuation of first subsequent postdiscontinuation therapy or death (whichever is earlier) | 0,54 (0,45-0,65) | 0,675 (0,558-0,816) | 0,757 (0,606-0,945) |
| 32861273 | BROCAD3 | Dieras | 2020 | 337 | 172 | carboplatin + paclitaxel + veliparib | carboplatin + paclitaxel + placebo | Control group could crossover to veliparib | 44 | 35,7 | time from randomisation to disease progression on first subsequent therapy or death from any cause | 0,71 (0,57-0,88) | 0,76 (0,6-0,96) | 0,95 (0,73-1,23) |
| 30345905 | PALOMA-3 | Turner | 2018 | 347 | 174 | palbociclib + fulvestrant | placebo + fulvestrant | ET, chemo | 5 | 44,8 | time from randomization to the end of the immediate subsequent line of therapy after disease progression | 0,58 (0,48-0,7) | 0,68 (0,56-0,84) | 0,81 (0,64-1,03) |
| 28578601 | OlympiAD | Robson | 2017 | 205 | 97 | olaparib monotherapy | single-agent chemotherapy | PARPi, platinum therapy | 8.2 | 14,5 | time from randomization to a second progression event or death after a first progression event | 0,58 (0,43-0,8) | 0,57 (0,4-0,83) | 0,9 (0,63-1,29) |
| 27502725 | TANIA | Minckwitz | 2014 | 247 | 247 | bevacizumab + chemotherapy | bevacizumab + chemotherapy | second-line progression-free survival alone |  | 15,9 | time from randomisation to disease progression or death on second-line treatment |  | 0,75 (0,61-0,93) |  |
| 24402830 | VITAL | Janni | 2014 | 75 | 37 | lapatinib + vinorelbine | lapatinib + capecitabine | crossover | 35 |  | time to second progression following crossover | 0,84 (0,53-1,35) |  |  |
